# Strain-activated light-induced halide segregation in mixed-halide perovskite solids

**DOI:** 10.1038/s41467-020-20066-7

**Published:** 2020-12-10

**Authors:** Yicheng Zhao, Peng Miao, Jack Elia, Huiying Hu, Xiaoxia Wang, Thomas Heumueller, Yi Hou, Gebhard J. Matt, Andres Osvet, Yu-Ting Chen, Mariona Tarragó, Dominique de Ligny, Thomas Przybilla, Peter Denninger, Johannes Will, Jiyun Zhang, Xiaofeng Tang, Ning Li, Chenglin He, Anlian Pan, Alfred J. Meixner, Erdmann Spiecker, Dai Zhang, Christoph J. Brabec

**Affiliations:** 1 Institute of Materials for Electronics and Energy Technology (i-MEET), Department of Materials Science and Engineering, Friedrich-Alexander-Universität Erlangen-Nürnberg (FAU), Martensstraße 7, 91058 Erlangen, Germany; 2 Institute of the Physical and Theoretical Chemistry, University of Tübingen, Auf der Morgenstelle 15, Tübingen, 72076 Germany; 3 Key Laboratory for Micro-Nano Physics and Technology of Hunan Province, College of Materials Science and Engineering, Hunan University, Changsha, 410082 People’s Republic of China; 4 Institute of Glass and Ceramics, Department of Materials Science and Engineering, University of Erlangen-Nuremberg, Martenstrasse 5, DE-91058 Erlangen, Germany; 5 Center for Nanoanalysis and Electron Microscopy (CENEM) & Institute of Micro- and Nanostructure Research (IMN), Interdisciplinary Center for Nanostructured Films (IZNF), Friedrich-Alexander-Universität Erlangen-Nürnberg, Cauerstraße 3, 91058 Erlangen, Germany; 6 Helmholtz-Institute Erlangen-Nürnberg (HI-ERN), Immerwahrstraße 2, 91058 Erlangen, Germany

**Keywords:** Materials science, Physics

## Abstract

Light-induced halide segregation limits the bandgap tunability of mixed-halide perovskites for tandem photovoltaics. Here we report that light-induced halide segregation is strain-activated in MAPb(I_1−x_Br_x_)_3_ with Br concentration below approximately 50%, while it is intrinsic for Br concentration over approximately 50%. Free-standing single crystals of CH_3_NH_3_Pb(I_0.65_Br_0.35_)_3_ (35%Br) do not show halide segregation until uniaxial pressure is applied. Besides, 35%Br single crystals grown on lattice-mismatched substrates (e.g. single-crystal CaF_2_) show inhomogeneous segregation due to heterogenous strain distribution. Through scanning probe microscopy, the above findings are successfully translated to polycrystalline thin films. For 35%Br thin films, halide segregation selectively occurs at grain boundaries due to localized strain at the boundaries; yet for 65%Br films, halide segregation occurs in the whole layer. We close by demonstrating that only the strain-activated halide segregation (35%Br/45%Br thin films) could be suppressed if the strain is properly released via additives (e.g. KI) or ideal substrates (e.g. SiO_2_).

## Abstract (web summary)

Mixed-halide perovskites are of interest for photovoltaic devices, but light-induced halide segregation obstructs bandgap tuning and is not fully understood. Here the authors study the effects of strain and iodide/bromide ratio on light-induced halide segregation in mixed-halide perovskites.

## Introduction

The development of stable perovskite polycrystalline films with varying bandgap is of great interest for perovskite-based optoelectronic devices such as solar cells, light-emitting diodes, lasers, and optical filters^1–7^. The bandgap of mixed-halide perovskites (APb(I_1−x_Br_x_)_3_, A = CH_3_NH_3_/NH_2_CHNH_2_/Cs) can be tuned from 1.5 to 2.3 eV by varying the Br content (here refers to X), which enables the fabrication of high-performance perovskite–silicon tandem and perovskite–perovskite tandem solar cells^8,9^. Moreover, bromide incorporation is also proven to increase the thermal stability of perovskites^10,11^.

As the ratio of bromide-to-iodide is changed in the precursor, colored perovskite films with varied bandgaps are feasibly tuned under dark^11–14^; however, under illumination, photo-induced halide migration leads to bandgap instability, mainly due to light-induced halide segregation (LHS)^12,15–24^. LHS leads to inhomogeneous bandgap across the perovskite layer, which severely limits the device performance under illumination.

To solve this issue, it is essential to reveal the driving force and all the relevant factors that affect this behavior in mixed-halide perovskites. Bischak et al. studied the nanoscale and dynamic imaging of LHS, revealing the polaron effect of photo-generated carriers on halide segregation^18^. Meanwhile, Draguta et al. claimed that bandgap reduction is the driving force for halide segregation rationalizing the non-linear intensity-dependency of LHS^20^. However, a unified picture remains under debate, given that contradictory results were reported in the literature. For instance, Tang et al. showed stable light emission in single crystals, and LHS is only occurring at the grain boundaries in polycrystalline films, whereas Mao et al. reported strong LHS in the single crystals^23,25^. The doping region without LHS was also inconsistently reported^15–25^. Furthermore, some studies showed that passivation or endotaxial matrices can effectively inhibit LHS, but this trend was not commonly observed by others^12,22,24,26,27^.

Here we report that LHS depends on both strain and composition in mixed-halide perovskites, which unifies many controversial results on LHS. We systematically study both free-standing and on-substrate single crystals of MAPb(I_1−x_Br_x_)_3_ with photoluminescence (PL) tracking. Device-relevant thin films are also studied, finding that LHS selectively occurs at grain boundaries in 35%Br polycrystalline films while it occurs across the whole layer in 65%Br films. These results prompt us to divide mixed-halide perovskites into three distinct regimes: LHS-free regime (e.g., 15%Br), strain-activated LHS regime (e.g., 35%Br), and intrinsic LHS regime (e.g., 65%Br). We demonstrate that the strain-activated LHS can be inhibited by releasing strain in the films via passivation strategies or lattice-match substrate, etc.

## Results

### Photoluminescence study of free-standing single crystals

**Fig. 1:**
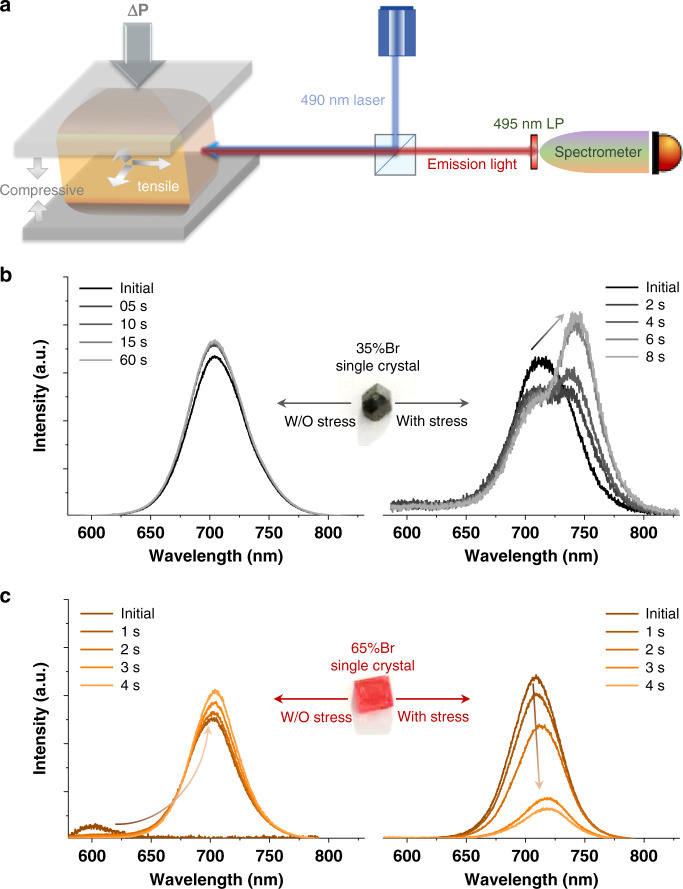
In-situ photoluminescence tracking of free-standing single crystals under uniaxial pressure. **a** The schematic diagram of in-situ PL measurement with external pressure. The excitation wavelength is 490 nm and the size of single crystal is around 5 mm in diameter. The laser intensity is approximately 100 mW cm^−2^. **b** The evolution of PL spectra for 35%Br single crystal under illumination without (left) and with external uniaxial pressure (right). The signal was collected immediately after we turned on the laser and “Initial PL” indicates the first measurement. The pressure was first applied to the single crystal under dark and the PL signal was collected immediately after the laser was turned on. **c** The evolution of PL spectra for 65%Br single crystal under illumination without (left) and with (right) external uniaxial pressure. The pressure is applied to 65%Br single crystal after the LHS process finishes under illumination and the “Initial PL” is collected after I/Br are fully segregated under illumination.

We first synthesized typical single crystals, such as MAPb(I_0.65_Br_0.35_)_3_ and MAPb(I_0.35_Br_0.65_)_3_, by using inverse-temperature reactive crystallization (see Methods)^14,28^. The composition of the single crystal is examined by energy dispersive X-ray spectra, showing a similar Br content to that in the precursor (Supplementary Fig. 1). The single crystal with a cleaved surface was mounted in a micrometer screw gauge to apply pressure perpendicular to the incident laser source (Fig. 1a and Supplementary Fig. 2). Under the illumination of 490 nm laser (~100 mW cm^−2^), 35%Br single crystal does not present LHS before applying the external stress, indicated by a stable PL at ca.704 nm, consistent with the bandgap of as-prepared MAPb(I_0.65_Br_0.35_)_3_ (Fig. 1b). A new peak appears at 740 nm after applying approximately 100 MPa uniaxial stress. The stress value is estimated from the product of Young’s modulus (15 GPa) and the displacement of the sample holder, which is close to the internal stress in perovskite polycrystalline film^29–31^. The second PL at 740 nm is assigned to the formation of MAPb(I_0.85_Br_0.15_)_3_. This phenomenon implies a mechanism of strain-activated LHS in 35%Br perovskite. We excluded the existence of secondary phases that would cause the second peak in our samples by X-ray diffraction (XRD) (Supplementary Fig. 3). Moreover, MAPbI_3_ does not show obvious PL change under the same pressure^32^, indicating a negligible change of bandgap (Supplementary Fig. 4). For 65%Br single crystals, an intrinsic LHS is observed before applying tress, evidenced by PL shift from 600 to 700 nm under illumination (Fig. 1c). The initial PL at 600 nm corresponds to the bandgap of as-prepared 65%Br while the final PL at 700 nm is attributed to the formation of MAPb(I_0.65_Br_0.35_)_3_. There might be a deviation for PL assignment due to the quantum confinement effect of these light-induced nanosize clusters^18,20^. The PL is further shifted from 700 to 720 nm after applying approximately100 MPa stress, implying that strain also influences the final segregation product. Previous studies of pressure’s effect on LHS are based on diamond-anvil cells to induce isotropic lattice compression in perovskite powders^17,31^. The LHS is found to be kinetically suppressed due to inhibited ion migration (the more compact of lattice, the less migration of halide). In contrast, we applied uniaxial pressure to the single crystal, resulting in compression and expansion of the lattice in two orthogonal axes considering a positive Poisson’s ratio. Therefore, it is derived that the tensile strain or complex lattice deformation, not the compressive strain, may be the key to activate LHS in the single crystals.

The strain-activated LHS in 35%Br crystal or intrinsic LHS in 65%Br crystal is reversible only when the laser is off (Supplementary Fig. 4). The halide-segregation metastable state is stabilized by laser illumination, which might be due to a polaron effect^18^. To obtain the limit of Br content with intrinsic LHS, we also studied three other single crystals with 45%Br, 55%Br, and 80%Br content. Here, 55%Br and 80%Br samples also show obvious intrinsic LHS, except for 45%Br sample; meanwhile, the kinetics of LHS become faster with more Br content, making it hard to track the real initial PL of 80%Br samples (Supplementary Fig. 5).

The strain-activated LHS might be related to the tetragonal–cubic phase transition in perovskites^33^. We examined PL evolutions at 100 °C which is above the transition point for 35%Br perovskite. The 35%Br sample still does not show LHS even if it goes through a phase transition, while 65%Br single crystal still presents strong LHS at high temperature (Supplementary Fig. 6).

### Photoluminescence imaging of on-substrate single crystals

Besides mechanical pressure on single crystals, strained perovskite can also be achieved by lattice-mismatched substrate^34^. Lattice mismatch at the interface with well-defined relative orientation can introduce strain into the softer material (here the perovskite)^35,36^.

**Fig. 2:**
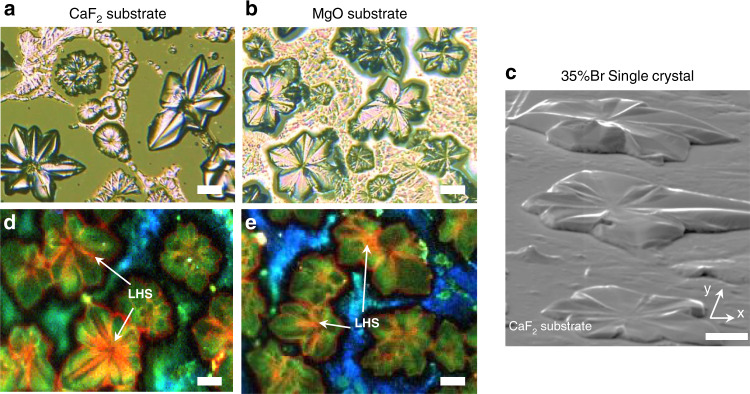
Hyperspectral imaging for on-substrate single crystals. **a**, **b** Wide-field optical images of 35%Br perovskite crystals fabricated on CaF_2_ and MgO single-crystal substrates via drop-cast method. The images were obtained under 50× object lens in reflection mode. **c** Electron backscatter image (70° tilted view) for EBSD analysis on perovskite crystals of 35%Br fabricated on CaF_2_ substrate. Scale bar: 10 μm. **d**, **e** Wide-field hyperspectral images of perovskite crystals fabricated on CaF_2_ and MgO single-crystal substrates. The LHS region is indicated by red color and the arrow. To excite the sample, 532 nm diode continuous-wave laser with approximately 100 mW cm^−2^ intensity is used. This image is constructed by superposing three narrow-band PL intensity mapping at 680 nm, 710 nm, and 740 nm (5 nm bandwidth). Here, 680 nm image is used as the background.

In this study, we fabricated 35%Br crystals on four types of single-crystal substrates (Mica, SiO_2_, CaF_2_, and MgO), using drop-cast method. We observed flower-shaped structures on CaF_2_/MgO substrates (Fig. 2a–c). To clarify whether this structure consists of adjacent multi-crystals or just one highly deformed single crystal, we characterized three different regions in one flower-like structure via electron backscatter diffraction (EBSD)^37^, as shown in Supplementary Figs. 7 and 8. EBSD can reveal the relative orientation between perovskite and CaF_2_ substrate. The results indicate a single-crystal character for this structure with <110> orientation close to *z*-axis and the <001> orientation close to the *y*-axis. Here the *z*-axis is defined as the direction perpendicular to the CaF_2_ substrate. Using the lattice constant of perovskite and CaF_2_, we further modeled the interface and found a large lattice mismatch (~10%) between perovskite and CaF_2_. The detailed analysis can be found in Supplementary Note 1. This large mismatch implies that tremendous strain energy is generated during crystal growth. This strain energy can be effectively reduced by crystal deformation, which will induce heterogeneously strained perovskites on CaF_2_ or MgO, leading to inhomogeneous LHS.

We then utilize hyperspectral imaging with different spectral windows to examine the LHS distribution in the deformed crystals. Since the PL peak is located at ca. 705 nm at the initial state while red-shift to 740 nm after halide segregation, we selected 710 and 740 nm with 5 nm bandwidth to image the inhomogenous LHS, marked in green and red colors, respectively. Under illumination of 532 nm laser, LHS is observed at the concave regions featuring a strong emission at 740 nm, indicated by red color, for both CaF_2_ and MgO substrate (Fig. 2d, e). Besides, for mica and SiO_2_ substrates, LHS is not observed in the crystals with a regular shape (Supplementary Figs. 9 and 10). The above phenomena can be attributed to the hetergeneous strain distribution due to large lattice mismatch at the interface.

### Characterization of halide segregation in polycrystalline films

We move on to study the effect of strain/composition on polycrystalline film since the most device-relevant samples are thin films on substrates. Different methods with/without antisolvent are used to fabricate the thin films on glass substrate (Supplementary Information). LHS is commonly observed in samples with Br content higher than 20% (20%Br-90%Br), although there is a large PL variation for the same composition when using different methods (Supplementary Fig. 11**)**.

**Fig. 3:**
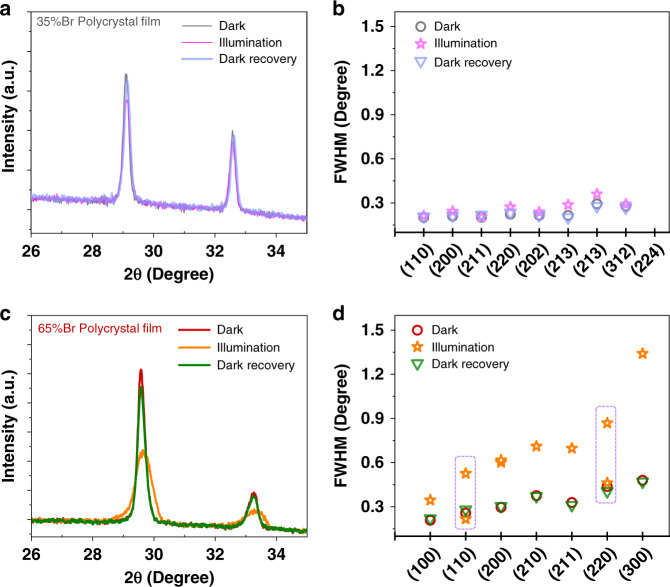
Structural characterization of polycrystalline thin films with dark–light–dark cycle. **a** XRD patterns for 35%Br perovskite film under dark and light. The light source is a white-light LED with an intensity of approximately 100 mW cm^−2^. The XRD of “Dark recovery” was measured after the LED light was off for approximately 3 min. **b** Analysis on full width at half maximum (FWHM) for the XRD peaks with dark–light–dark cycle. XRD peak change (**c**) and FWHM change (**d**) for 65%Br perovskite film.

To make a comparison with the single-crystal experiments above, we examined two typical perovskite films (i.e., 35%Br and 65%Br) fabricated through antisolvent method. We first characterized the structural change of 35%Br and 65%Br thin films by in-situ XRD using a home-made LED light source with dark–light–dark cycle (Supplementary Fig. 12). For 35%Br film, we did not observe a distinct variation of XRD patterns under 100 mW cm^−2^ illumination except for a lower peak intensity (Fig. 3a, b); however, 65%Br film presents distinguishable peak broadening in XRD patterns, accompanied by a large intensity drop (Fig. 3c, d). All the peaks recover to their original positions when the samples were stored under dark for approximately 3 min. The reversibility of XRD patterns is consistent with the reversible PL change under dark **(**Supplementary Fig. 4)^19,21^. To further validate such differences, we characterized 35%/50%/65%Br samples with varying thickness under 100 or 150 mW cm^−2^ illumination. The XRD peaks of all the 35%Br films only show small broadening, but the perovskite samples with Br content over 50% presented clear peak splitting (Supplementary Fig. 13). The degree of peak splitting is also found to be orientation-dependent.

Although both films show strong PL splitting under laser illumination, 35%Br thin films show much less structural change than 65%Br samples. Combined with the above single-crystal experiments, we speculate that LHS only occurs at some highly strained regions in 35%Br thin film while it occurs in the whole layer of 65%Br sample.

### Local observation of halide segregation in polycrystalline films

To verify the above conjecture, we utilized shear-force scanning probe microscopy (SPM) combined with confocal PL to obtain the local LHS in perovskite polycrystalline films^38^. By scanning the perovskite film with a sharp gold tip positioned in the focus of a 532 nm laser, we could directly correlate the sample topography with PL signals at the same location with a resolution around 200 nm. We investigated two typical samples on a glass substrate, namely 35%Br and 65%Br perovskite polycrystalline films with an averaging grain size of 500 nm (Supplementary Fig. 14). The topography image clearly shows grain boundaries, which enables us to selectively track the PL spectra at the grain center and grain boundary.

**Fig. 4:**
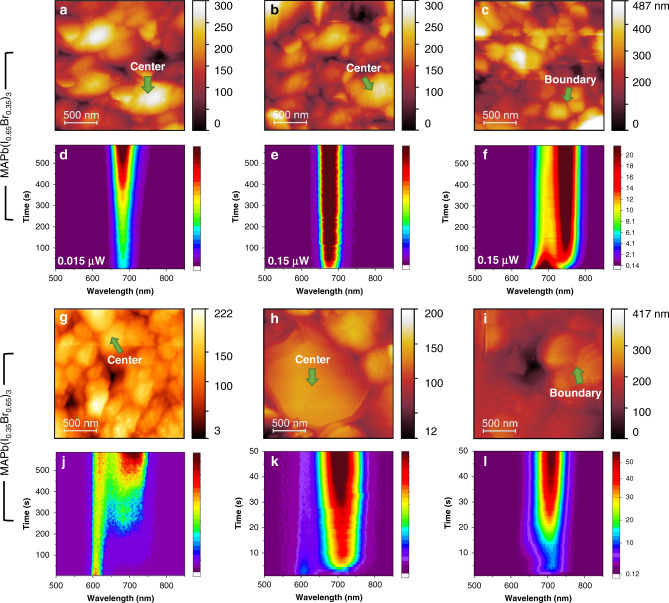
Local LHS study of polycrystalline thin films. **a**–**c** Shear-force topographic images of a 35%Br film collected from different regions. **d**–**f** The PL spectra evolution at grain center and grain boundaries of 35%Br perovskite films, which were taken with 1 s interval. The positions where the spectra were taken are indicated by the green arrows showing in a, b, and c corresponding to (**a**), (**b**), and (**c**), respectively. To excite the sample, 532 nm diode continuous-wave laser is used. The laser power of 0.01 μW, 0.1 μW, and 0.1 μW is used in (**d**), (**e**), and (**f**), respectively. **g**–**i** Shear-force topographic images of 65%Br films collected from different regions. **j**–**l** The PL evolution at grain center and grain boundaries of 65%Br perovskite which was taken with 1 s interval, corresponding to (**j**), (**k**), and (**l**), respectively. The laser power of 0.015 μW, 0.15 μW, and 0.15 μW are used in (**j**), (**k**), and (**l**), respectively. To excite the sample, 532 nm diode continuous-wave laser is used.

As shown in Fig. 4a, b, d, e, when the 532 nm laser is focused on the grain centers of 35%Br films on glass substrates, the PL peak position remains stable. The slowly increased PL intensity is attributed to light-induced defects healing^39,40^. In contrast to the grain centers, the grain boundaries show evident LHS (Fig. 4c, f). For 65%Br film, both the grain centers and the grain boundaries show PL splitting, indicating halide segregation across the whole film (Fig. 4g–l). Hence we conclude that LHS is not an intrinsic property in 35%Br perovskite, clearly differing from 65%Br perovskite. Besides, with increased illumination, the PL evolution becomes faster. Note that the initial peak spectra of 65%Br become hard to track when laser power is increased from 0.01 μW **(**Fig. 4k) to 0.1 μW **(**Fig. 4l), due to a fast PL evolution with higher laser power. This proportionality between laser power and LHS kinetics can be interpreted by a nucleation process of iodide-rich domains triggered by polarons-with stronger illumination, a faster nucleation/growth rate is expected^20,24^.

Combined with the results on single crystals, the local LHS at grain boundaries in 35%Br films is ascribed to local strain at these regions. To support our scenario of strain inhomogeneity in perovskite films, we first demonstrate an averaging 0.3% strain in the thin films using Williamson–Hall plot^15,41,42^. This value is much larger than that in single crystals (~0.0004%) (Supplementary Fig. 15). Compared with single crystals, local strain characterization, via confocal Raman, further implies a pronounced strain inhomogeneity in the thin films (Supplementary Fig. 16). A detailed strain analysis is presented in Supplementary Note 2.

### Suppression of light-induced halide segregation

**Fig. 5:**
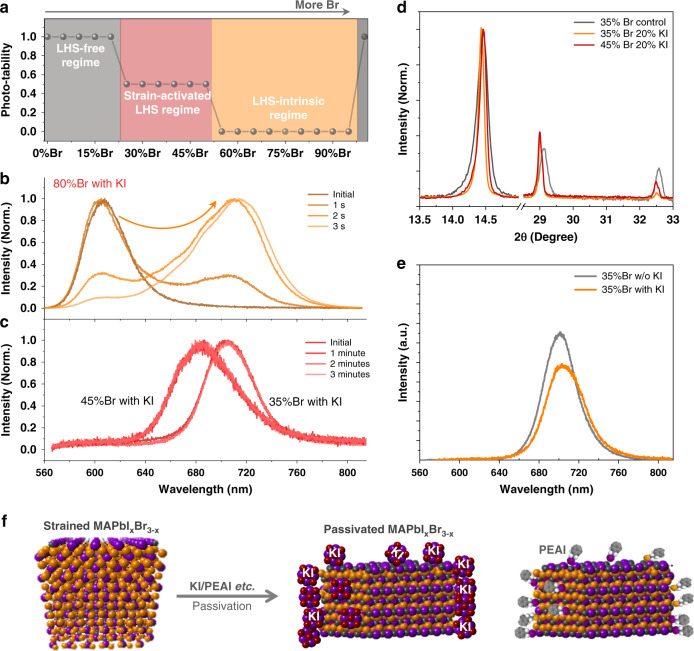
Suppression mechanisms of LHS in polycrystalline thin films. **a** The schematic illustration of three distinct regimes for LHS in mixed-halide perovskites MAPb(I_1-x_Br_x_)_3_. With more Br, MAPb(I_1-x_Br_x_)_3_ goes through from LHS-free regime (e.g., MAPb(I_0.85_Br_0.15_)_3_) to strain-activated LHS region (e.g., MAPb(I_0.65_Br_0.35_)_3_) and then to LHS-intrinsic region (e.g., MAPb(I_0.35_Br_0.65_)_3_). **b**, **c** PL evolution for the mixed-halide perovskite films (35%Br/45%Br/80%Br) with 20 mol.% KI passivation. The excitation wavelength is 490 nm and the laser intensity is approximately 100 mW cm^−2^**. d** The XRD patterns of mixed-halide perovskites with and without KI passivation. Note that the XRD patterns of samples with KI additive are aligned with the XRD of the control samples. **e** Initial PL spectra for 35%Br perovskite films without and with 20 mol.% KI, showing a similar PL intensity. The excitation wavelength is 490 nm and the laser intensity is approximately 100 mW cm^−2^. **f** The schematic of surface passivation by KI/PEAI to release the strain in perovskites.

The strain-activated and composition-dependent LHS prompts us to divide mixed-halide perovskites into three distinct regions depending on Br concentration: (i) LHS-free region (e.g., MAPb(I_0.85_Br_0.15_)_3_), (ii) strain-activated LHS region (e.g., MAPb(I_0.65_Br_0.35_)_3_), and (iii) intrinsic-LHS region (e.g., MAPb(I_0.35_Br_0.65_)_3_). We expect that only the strain-activated LHS could be suppressed by grain-boundary passivation, as is shown in the schematic diagram (Fig. 5a).

We close by exploring the passivation strategy and its mechanism to suppress LHS in mixed-halide perovskite films. KI or phenethylammonium iodide (PEAI) is added to the precursor solution because these K^+^/PEA^+^ mainly stay at the grain boundaries due to their incompatible size. The PL spectra show that excess 20 mol.% of KI is capable of stabilizing 35%/45%Br perovskites under illumination. As is expected, 70%/80%Br perovskites cannot be stabilized even with excess 40 mol.% KI (Fig. 5b, c and Supplementary Fig. 17). Similar to KI, 35%Br and 45%Br perovskite films with excess PEAI also show improved stability (Supplementary Fig. 18). With excess KI at grain boundaries, less broadening is observed in XRD patterns compared with samples without KI (Fig. 5d). The reduced broadening is normally related to strain release, size increase, or defects reduction. In our samples, the grain size is very similar for perovskite films with/without KI (Supplementary Fig. 14). Even if we assume the grain size increases from 200 to 500 nm, there will be only 0.02° reduction, far below the total reduction of 0.07°. PL spectra do not strongly support defects reduction by KI because the PL intensity is not enhanced by KI additive in our case (Fig. 5e). We conclude that strain release is the main mechanism for the narrowed XRD patterns, as shown in Fig. 5f.

## Discussion

In terms of thermodynamic phase stability under dark, Br can be stably mixed with iodide in the whole composition space of MAPb(I_1-x_Br_x_)_3_. Under optical illumination, the formation of iodide-rich phases is observed, which is rationalized by bandgap reduction and polaron effect^18,20^. Although the light-induced halide segregation (LHS) is commonly recognized, it is still regarded as an intrinsic property in mixed-halide perovskites^12,15,22–27,43^. Our results elaborate on the incomplete understanding of LHS by unveiling the effects of strain on LHS. From the free energy point of view, it demands a larger driving force for the unstrained perovskite to induce halide segregation (Supplementary Note 3). Thus, two distinct regimes should be recognized: strain-activated LHS regime and intrinsic-LHS regime. We conclude that without the synergetic effect of strain, the driving force of bandgap reduction and polaron is not enough to induce halide segregation within the strain-activated LHS regime. We further demonstrate that only the perovskites within the first two regimes can be utilized to fabricate photo-stable perovskite film for long-term device applications. For example, LHS is strain-activated for Br content below 50%, which can be inhibited by passivation and ideal substrate (e.g., SiO_2_). The regime definition for strain-activated LHS is also affected by cation substitution since LHS is found to be inhibited in CsPb(I_0.35_Br_0.65_)_3_ (Supplementary Fig. 19).

In the polycrystalline films, strain normally arises during crystal growth via spin-coating/drop-casting/evaporation, except for an ideal epitaxial growth^37,44–46^. More importantly, strain prefers to concentrate at the grain boundary due to the effects of gap closure between two oriented grains, defects pile-up (e.g., dislocation, Schottky defect), etc.^35,42,44,45,47,48^. The great impact of strain on physical properties has also been demonstrated in diverse material systems^47,49,50^. Our work unifies the debate over LHS in mixed-halide perovskites by revealing the effect of strain on halide segregation, which could potentially guide the researcher to prepare more efficient and stable tandem photovoltaics or LEDs.

## Methods

### Single crystal preparation

All the chemicals are purchased from Sigma-Aldrich unless otherwise stated. For perovskite single crystal synthesis, we followed the previous work by Zhang et al.^14^. First, we prepared 0.7 M MAPbBr_3_ DMF precursor for 20 mL stirring at 30 °C for 30 min and 1.3 M MAPbI_3_ GBL precursor for 20 mL stirring at 80 °C for 60 min. The desired mixed-halide solution was obtained by mixing the prepared solution together and filtered to get a clear solution (PTFE, 0.45 μm). All the solutions were kept on a programmable hotplate starting from 30 to 80 °C at a rate of 5 °C/h. The temperature of the hotplate was then slowly increased to 90 °C within 48 h (no cover on the vials). Since precipitates could be formed in mixed-halide perovskite solution once the solution cools down to room temperature, the transfer of solution needs to be done quickly. When the solution reached an oversaturation state, lots of crystal seeds were formed. We picked up a 2 mm-size perovskite crystal with a hot spoon (very important) into another fresh solution kept at 90 °C. Once the crystal was over 5 mm, we used tissues to wipe off the surface of single crystals. The other methods and raw data can be found in the Supplementary materials.

### Photoluminescence characterizations

The in-situ PL measurement was finished by a home-made setup equipped with a 490 nm diode laser (Oxxius) and a modified micrometer to apply external stress to the sample. The stress (σ) was roughly estimated by multiplying Young’s modulus (Y) by the relative displacement (δL/L). With a fixed stress ~100 MPa on the cleaved single crystal, we opened the laser shutter to measure the PL evolution. The luminescence was collected in back-scattering geometry, dispersed by an iHR320 monochromator (Horiba JobinYvon), and recorded with a Peltier-cooled Si charge coupling device (CCD; Synapse, Horiba Jobin-Yvon). The PL measurement at 100 °C was realized by using a heated graphite stage where the sample is mounted on. The superposed PL image is obtained by using IMA^™^ hyperspectral microscopy platform, which delivers the PL image with high spectral and spatial resolution.

### Perovskite film fabrication

The glass substrates were washed in sequence in acetone and isopropanol with sonication for 60 min. These substrates were treated with ozone plasma for 2 min before transferring to the glovebox. Then, 1 M MAPbBr_3_ DMF precursor, 1 M MAPbI_3_ DMF precursor, and 1 M MAPbI_3_ GBL precursor were prepared by stirring at 30 °C, 60 °C, and 80 °C, respectively; 1 M KI and PEAI were dissolved in DMSO by stirring at 30 °C. The desired mixed-halide solution with/without KI/PEAI additives were prepared by mixing the resulting solution with different volume ratios. For 10 mol.%KI passivation, the solution is prepared by adding 0.1 mL KI into 1 mL perovskite-precursor solution. In case otherwise stated, the composition of 35%Br, etc. refer to the composition in our precursor solution. For 100 and 250 nm-thick perovskite film, we used 200 rpm/3 s→2000 rpm/0 s and 2000 rpm/5 s→5000 rpm/40 s, respectively; then we drop-cast 150 uL antisolvent of chlorobenzene (CB) onto the film before the end of spin-coating process. CB is dropped at 10 s/20 s/20 s/30 s/30 s/30 s for MAPbI_3_/35%Br/45%Br/65%Br/75%Br/MAPbBr_3_, respectively, before the end of spin-coating. The films were annealed at 70 °C for 1 min and 110 °C for 20 min, to form shiny perovskite films. For single crystal, PbI2 is purchased from MERCK (99.99%) while it is from TCI for fabricating thin film. The organic salt (MAI/MABr) is from Xi’an Baolaite (p-OLED). All of the solvents are from MERCK unless otherwise stated.

### Confocal photoluminescence under shear-force scanning probe microscope

Combined optical spectroscopy and topography measurements have been conducted using a home-built parabolic mirror-assisted confocal optical microscope setup, expanded with a shear-force SPM function. A continuous-wave mode 532 nm laser (QIOPTIQ, NANO 250-532 max) was used as an excitation source. A radially polarized beam was used to reach a diffraction-limited focus, which was done by guiding the linearly polarized beam through an expanding telescope followed by a lambda-half plate mode converter into a radially polarized beam. A large opening angle parabolic mirror (numerical aperture of 0.998) was used to focus the optical beam on the sample. The high numerical aperture results in diffraction-limited optical resolution. Sample-emitted optical signals were then cleared from elastic scattering using two optical 1 filters (12 optical density in total) and detected by an avalanche photo diode (Single Photon Counting Module SPCM-AQR-14; Perkin Elmer) for optical imaging. Simultaneously, liquid nitrogen-cooled CCD coupled to a spectrometer (SpectraPro 300i; Acton Research) was used to collect PL spectra with a 150 grooves grating. A chemically etched gold-tip mounted to quartz tuning fork using UV curable glue was used to collect topography. The tuning fork approached the sample through a gap at the top of the parabolic mirror and is then placed in the focus to ensure position correlation between the optical and topographical signals. A constant tip–sample distance was kept by monitoring the phase shift using an Ametek 7270 DSP lock-in amplifier and regulating the distance with an RHK-SPM100. Obtaining optical and topographic images is done by raster scanning the sample through the focus. Then selective spots were chosen for the acquisition of optical spectra. Time traces have been collected at 1 s integration times with a 0.05 s dead time in between two spectra.

### Confocal Raman measurement

Raman spectra were acquired using an iHR 320 Horiba monochromator combined with a Sincerity UV-VIS CCD camera (2048-70, 14 mm). The light source is a Coherent Sapphire SF single frequency 100 mW, 488 nm CW laser. The setting is equipped with an OptoSigma PAL-50-L objective (×50, numerical aperture 0.42, working distance 20.5). An extended description of the equipment can be found in our previous paper^51^. The gratings have been calibrated using the peak positions of crystalline calcite (CaCO_3_).

### X-ray diffraction and electron backscatter diffraction

X-ray powder diagrams were recorded on an X’PertMPD PRO from PANalytical equipped with a ceramic tube (Cu anode, λ = 1.54060 A), a secondary graphite (002) monochromator, and a RTMS X’Celerator detector, and operated in BRAGG-BRENTANO geometry. For in-situ light illumination, we assembled a mini-LED light source with a cooling fan. The light intensity is calibrated by a power detector using 512 nm as the wavelength (Thorlabs, S120VC). The strain analysis is based on a software of X’Pert HighScore Plus provided by PANalytical company.

EBSD measurements are performed using a FEI Helios NanoLab 660 DualBeam FIB instrument equipped with an Oxford Symmetry Detector operated below 20 kV acceleration voltage and a beam current of 1.6 nA. Acceleration voltages above 20 kV and higher beam currents lead to a fast degradation of the perovskite accompanied by the appearance of PbI_2_ signal. For the chosen illumination conditions, a subsequent EBSD map still does not show any PbI_2_ signal, demonstrating that the results are not influenced by beam damage.

## Supplementary information

Supplementary Information

Peer Review File

Supplementary Data 1

## Data Availability

Data that support the findings of this study are available in separate Supplementary Data files in the Supplementary Information section. All other relevant data are available from the corresponding authors upon reasonable request.
