# Supplementary material for: Strain-activated light-induced halide segregation in mixed-halide perovskite solids
Source: Nat Commun. 2020 Dec 10;11:6328. doi: 10.1038/s41467-020-20066-7 (PMC7730187; doi:10.1038/s41467-020-20066-7)
Supplement: Supplementary file 1 — Supplementary Information [file 41467_2020_20066_MOESM1_ESM.pdf]

Supplementary Information

Strain-activated light-induced halide segregation in mixed-halide perovskite solids

Y. Zhao et al.
Corresponding to:
yicheng.zhao@fau.de (Y.Z.)

Supplementary Note 1

For EBSD analysis, the pronounced topology of the perovskite flowers, in combination with the beam sensitivity of the perovskite only allows us to obtain Kikuchi patterns without fine structures for the perovskite flower shown in **Supplementary Figure 8**a. These patterns disable us to analyze the local strain from the fine structures (distortion and broadening). But fortunately, these data enable us to derive the relative orientation between perovskite and the single-crystal substrate (CaF_2). Crystallographic pole figures are then extracted from the Kikuchi patterns. The pole figure of perovskite shows a single-crystalline character with <110> orientation close to z-axis and <001> orientation close to y-axis. For CaF_2, it shows <111> orientation close to z-axis and <110> orientation close to y-axis. Combined with the lattice constant (Pb-I: 0.3 nm, I-I: 0.6 nm; Ca-F: 0.24 nm, Ca-Ca: 0.39 nm) and CIF files, we depict the top view and the side view of this lattice-mismatched interface (**Supplementary Figure 7f & Supplementary Figure 8c**). Z-direction indicates the direction perpendicular to CaF_2 substrate here. The CIF files are attached in the raw data file.

Supplementary Note 2

The strain in perovskite thin film was characterized by Williamson-Hall plot. A linear function *Bcos(θ)* of *sin(θ)* can be approximated to fit the strain ε based on the formula below:

$$\mathrm{B}\,cos(\theta) = \frac{0.9\lambda}{D} + 4\varepsilon\sin(\theta) + B_0\sin(\theta) \quad (1)$$

where B is the peak broadening from Lorenz fitting, θ is the diffraction angle, D is the grain size, λ is 0.154 nm and B_o is the instrumental broadening. An averaging strain of 0.3% and 0.0004% is obtained for 65%Br thin film and 65%Br single crystal, respectively (**Supplementary Figure 15a/c**). The analysis for other single crystals (*i.e.* $MAPbI_3$/$MAPbBr_3$) also shows a trivial strain. We also note that 35%Br presents singular peak broadening caused by its tetragonal phase-induced broadening. The orientation-dependent strain from 0.2% to 0.4% is obtained by directly using Formula (1) (**Supplementary Figure 15b**). With grain size D=500 nm, the size-related broadening is 0.016 ° for diffraction angle at 14 ° and strain of 0.27% is obtained by subtracting the broadening of single crystal (0.07 °) and size effect (0.016 °) from the total broadening (0.17 °). Single crystal is referred to as strain-free reference and equivalent to instrumental broadening here. The Schottky defect is not included for XRD broadening because the derived strain value under this assumption is close to Williamson-Hall analysis.

To further acquire the information of local strain, we use confocal Raman to analyze the strain inhomogeneity due to its sensitivity to local strain variation [1,2]. Here a 488 nm laser is used to give an optical resolution of approx. 300 nm. Since perovskite is not Raman-active at room temperature, we utilize the laser-induced decomposition product PbI_x to reflect the local strain in perovskite thin film, considering the interaction between PbI_x and the adjacent perovskite[1,3,4]. Two peaks are appearing at 82.9/136.1 cm^{-1} and 83.2/145.2 cm^{-1} for single crystal and polycrystal film, respectively (**Supplementary Figure 16a/c**). Compared with single crystal, the A_{1g} Raman spectrum of PbI_x in the polycrystalline film is shifted to higher wavenumbers and broadened from 5.8 to 9.6 cm^{-1}. Furthermore, the Raman spectra show a larger variation in the polycrystal film (between 138 cm^{-1} and 148 cm^{-1}) than single crystal ((**Supplementary Figure 16b/d**)). These results imply complex strain inhomogeneity in polycrystal film, consistent with a recent work by T. Jones *et al.* and S. Jariwala *et al.* demonstrating local strain in mixed-halide perovskite films[5,6]. Here $MAPbI_3$ is selected instead of 35%Br sample to avoid the signal interference from laser-induced halide segregation during measurement. The local halide segregation at the boundaries in 35%Br sample will affect the intrinsic strain distribution in polycrystal film.

Supplementary Note 3

To discuss the mechanism of strian-activated LHS, we use a simplified model to analyze the thermodynamics of mixed-halide perovskite with and without lattice deformation, or internal strain, as follows:

Without lattice deformation:

$$\Delta \mathrm{F} = F_{seg.} - F_{mix}$$

$$= \left(U_{seg.} - U_{mix}\right) - k_B T N_0 [x_{\mathrm{Br}} ln x_{\mathrm{Br}} + (1 - x_{\mathrm{Br}}) \mathrm{ln}(1 - x_{\mathrm{Br}})] + N \Delta \mathrm{E}_g + N\, \Delta \mathrm{E}_p + (E^{s1.}_{seg.} - E^{s1.}_{mix})$$

$$= \Delta U - T \Delta \mathrm{S} + N \Delta \mathrm{E}_g + N\, \Delta \mathrm{E}_p + (E^{s1.}_{seg.} - 0)$$

With lattice deformation:

$$\Delta \mathrm{F} = F_{seg.} - F_{mix}$$

$$= \left(U_{seg.} - U_{mix}\right) - k_B T N_0 [x_{\mathrm{Br}} ln x_{\mathrm{Br}} + (1 - x_{\mathrm{Br}}) \mathrm{ln}(1 - x_{\mathrm{Br}})] + N \Delta \mathrm{E}_g + N\, \Delta \mathrm{E}_p + (E^{s2.}_{seg.} - E^{s2.}_{mix})$$

$$= \Delta U - T \Delta \mathrm{S} + N \Delta \mathrm{E}_g + N\, \Delta \mathrm{E}_p + \Delta E^{s2}$$

From experimental results:
$$\Delta \mathrm{E}_g = (0.9 x_{\mathrm{Br}} - 0.25 x_{\mathrm{Br}}^2) \cdot N$$

Where U, S, T is formation energy of perovskite, entropy, temperature respectively; N_0 is lattice cell number; N is carrier density, $\Delta \mathrm{E}_g$ is the energy reduction by the funneling of photo-carriers to I-rich clusters; $\Delta \mathrm{E}_p$is the reduction of polaronic energy due to halide segregation, ΔE^s is the gain of strain energy around I-rich/Br-rich interfaces due to halide segregation.

As a function of Br concentration (x_{Br}), the sum of the first two terms is positive, while the third and the fourth term is negative for $x_{\mathrm{Br}} > 20\%$ [1,2]. The last term is positive considering the lattice mismatch between I-rich and Br-rich perovskite. Without lattice deformation (*e.g.* free-standing single crystals), the strain energy is close to 0 at the mixed state; however, it becomes non-trivial for the strained perovskites at the mixed state.

The halide segregation will occur if $\mathbf{\Delta F < 0.}$ Only the 3rd and the 4th term are the driving force of halide segregation because the photo-carriers can funnel into the iodide-rich clusters to minimize the total free energy. We can derive that the gain of strain energy caused by halide segregation is lower for the strained perovskite ($\Delta E^{s2} = E^{s2.}_{seg.} - E^{s2.}_{mix}$), compared with the unstrained perovskite ($E^{s1.}_{seg.}$). Thus, it is equivalent to a lower driving force for the strained perovskites to induce halide segregation.

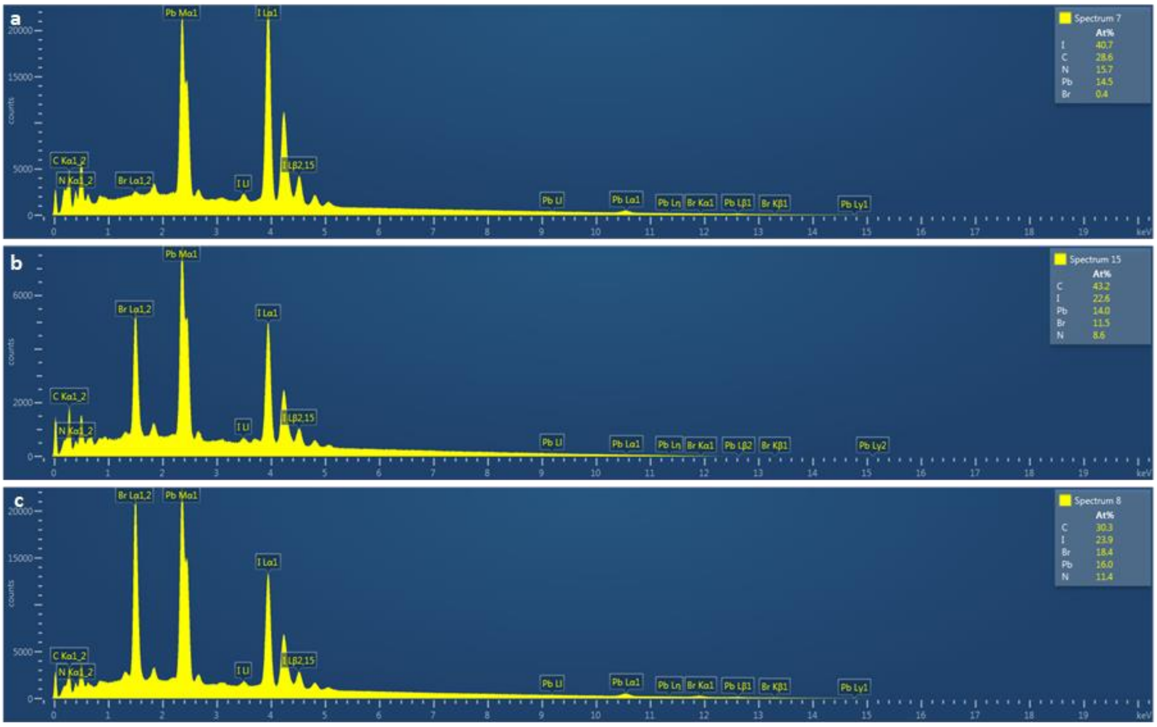

Supplementary Figure 1. Composition analysis of single crystal $MAPbI_3$ **(a)**, $MAPb(I_{0.65}Br_{0.35})_3$ **(b)**, $MAPb(I_{0.55}Br_{0.45})_3$ **(c)** *via* energy dispersive X-ray spectra (EDX). EDX result shows 1%Br, 33.5%Br and 43.5%Br for the three samples respectively.

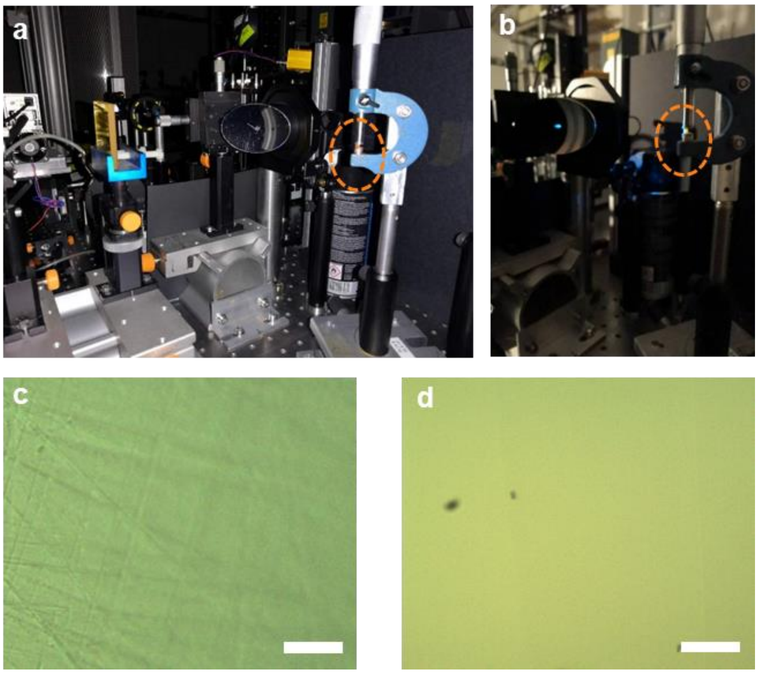

Supplementary Figure 2. The setup for *in-situ* PL measurement with external pressure under dark **(a)** and illumination **(b)**. Optical image of the cleaved surface for 35%Br single crystal **(c)** and 65% single crystal **(d).** The scale bar is 10 μm.

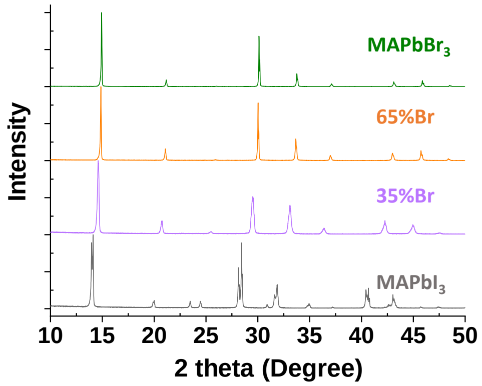

Supplementary Figure 3. X-ray diffraction patterns of perovskite single crystals from $MAPbI_3$ to $MAPbBr_3$ using inverse-temperature reactive crystallization. The samples were grinded into powder before measurement.

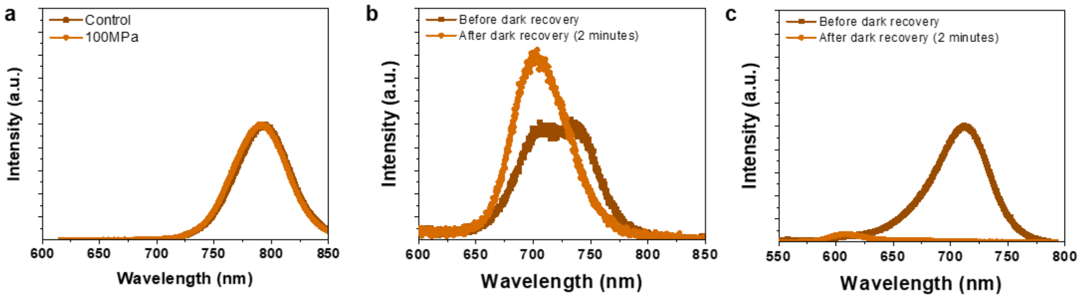

Supplementary Figure 4. a The PL evolution for $MAPbI_3$ single crystal at room temperature under ~100 MPa pressure. **b** The PL spectra of 35%Br single crystal with halide segregation (gray line) and after 2 minutes' dark recovery (pink line) without external stress. **c** The PL spectra of 65%Br single crystal with halide segregation (gray line) and after 2 minutes' dark recovery (pink line).

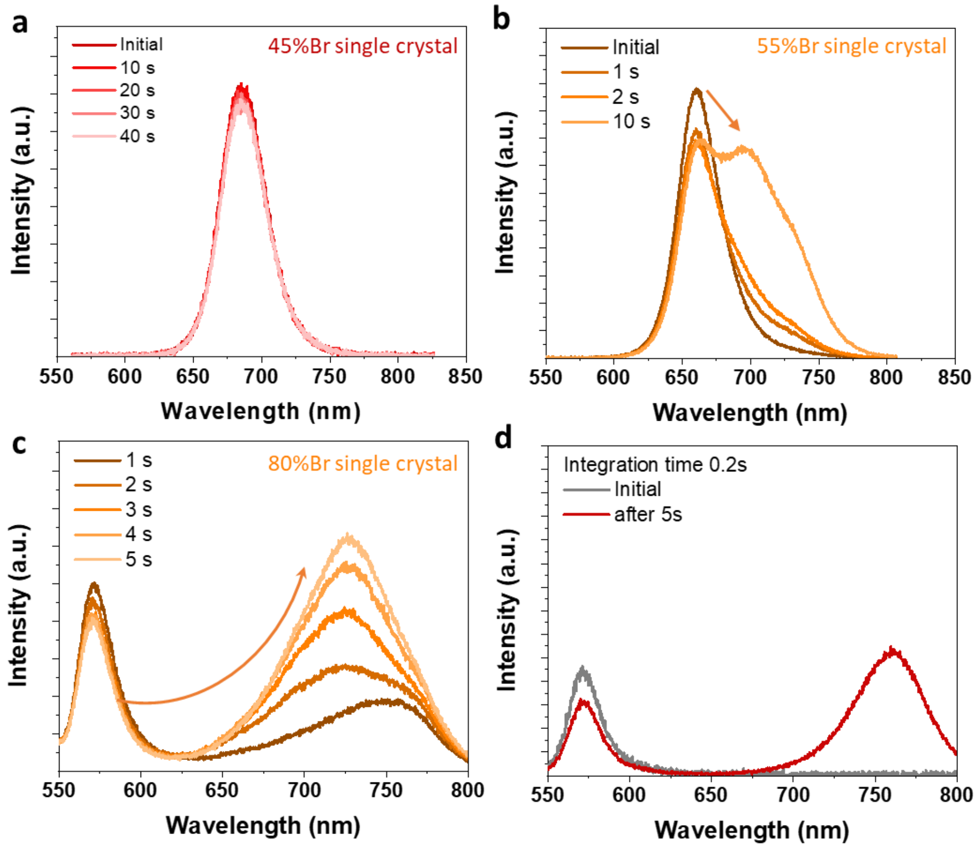

Supplementary Figure 5. a PL spectra of the 45%Br perovskite single crystal under 490 nm laser illumination (~100 mW cm^{-2}) with 1 s integration time for each measurment. **b** PL spectra of the 55%Br perovskite single crystal under 490 nm laser illumination (~100 mW cm^{-2}) with 1 s integration time for each measurment. **c** PL evolution for 80%Br single crystal under illumination of 490 nm laser (~100 mW cm^{-2}) with 1 s integration time for each measurment. **d** PL evolution for 80%Br single crystal under illumination of 490 nm laser (~100 mW cm^{-2}) with 0.1 s integration time for each measurment.

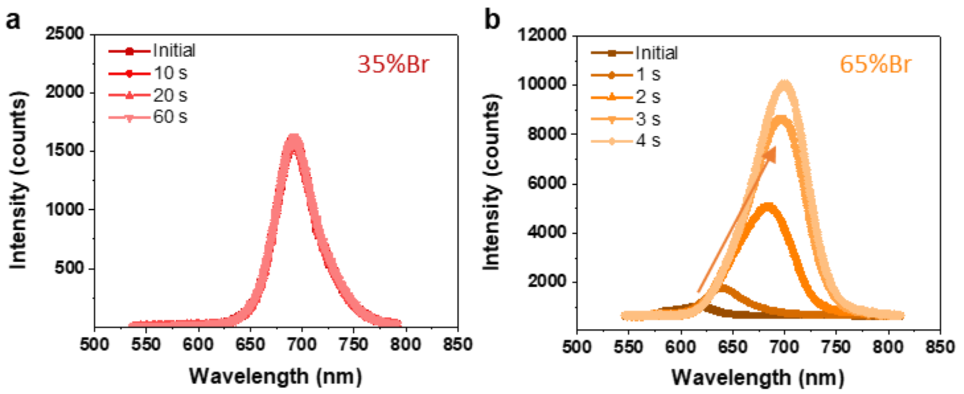

Supplementary Figure 6. a The PL evolution for 35%Br single crystal at 100 °C under continuous illumination of 490 nm laser with 1s integration time. **b** The PL evolution for 65%Br single crystal at 100 °C. The integration time for each measurement is 1 second.

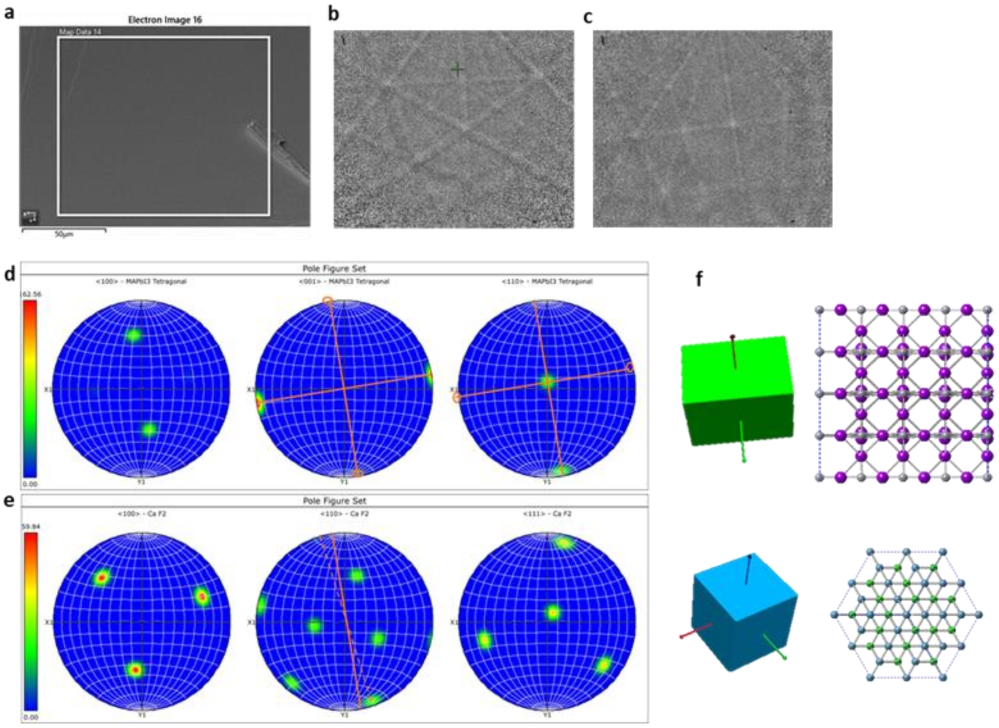

Supplementary Figure 7. a SEM micrograph (70 ° tilted view) of free-standing single crystal 35%Br perovskite with the indicated region for subsequent EBSD analysis. **b** Kikuchi pattern of 35%Br single crystal as derived. **c** Kikuchi pattern of on-CaF_2 35%Br single crystal. **d** Pole figure of on-CaF_2 35%Br crystal. Note in Figure d: from the Kikuchi pattern, it is almost impossible to distinguish between (110) and (002) lattice planes due to a high similarity between the patterns of the two planes, which is an intrinsic and recognized character for tetragonal perovskite phase. **e** Pole figure of the CaF_2 substrate. The orange solid line represents one of <001> poles of perovskite while the dashed line represents one of <110> poles of CaF_2. **f** Top-view crystal structure of perovskite and the substrate CaF_2. The top left cuboid represents the unit-cell orientation of tetragonal perovskite. The bottom left cubic represents the unit-cell orientation of cubic structure CaF_2.

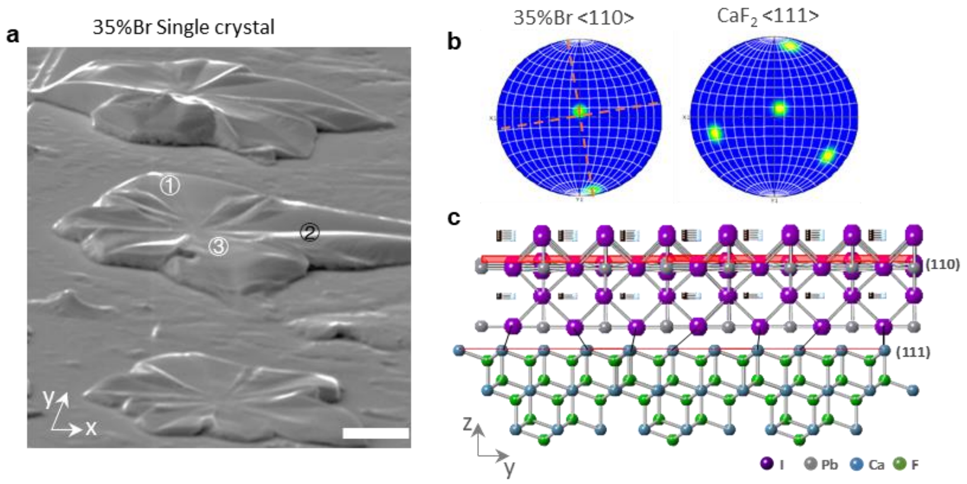

Supplementary Figure 8. a Electron backscatter image (70 ° tilted view) for EBSD analysis on perovskite crystals of 35%Br fabricated on CaF_2 substrate. Three different positions are characterized, marked by 1/2/3. **b** Pole figures of the substrate CaF_2 and 35%Br perovskite derived from Kikuchi patterns of EBSD, based on the averaging value of point 1/2/3. Scale bar: 10 μm. **c** Side-view crystal structures of CaF_2/35%Br perovskite oriented at <111>/<110> in z-axis and <110>/<001> in y-axis.

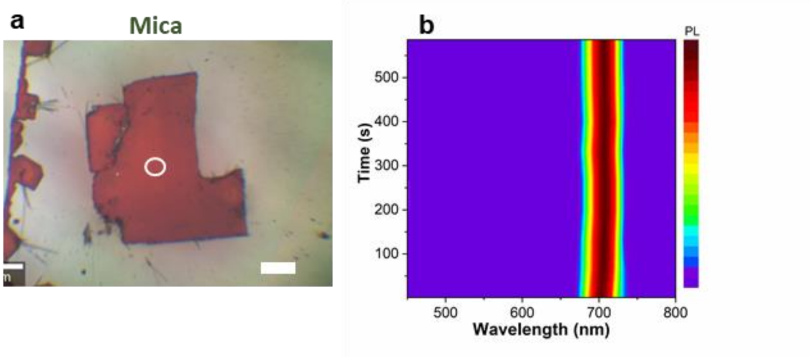

Supplementary Figure 9. Optical image **(a)** and PL evolution **(b)** of 35%Br crystals on Mica substrate. 532 nm laser with 0.1 μW power is used here. The integration time for each measurement is 5 seconds. The scale bar is 10 μm.

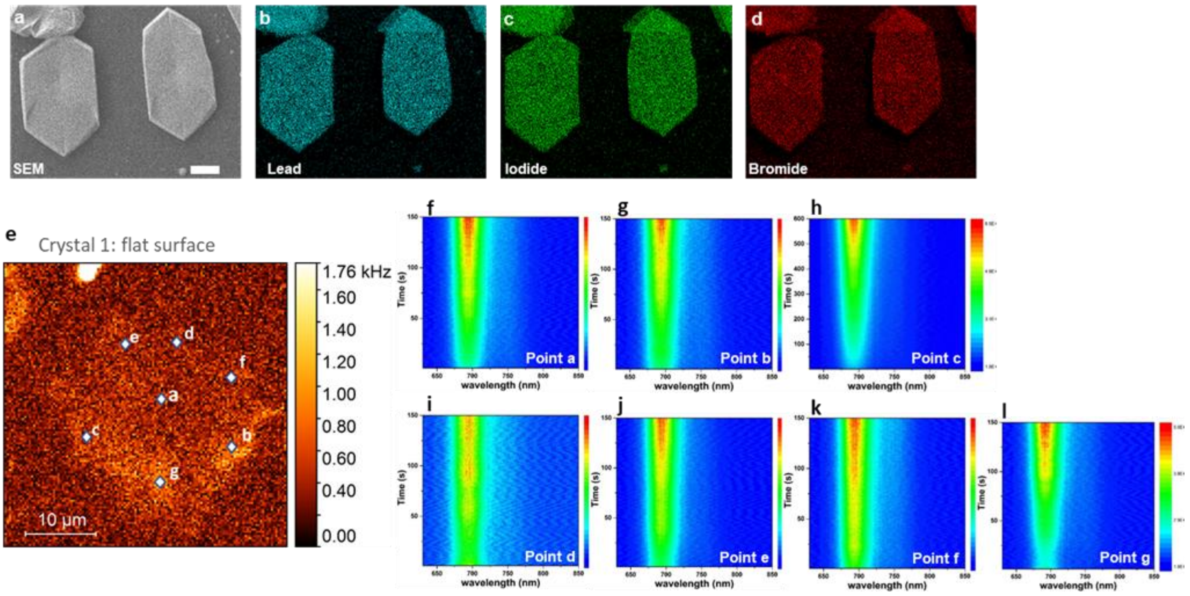

Supplementary Figure 10. a Scanning electron microscope of 35%Br perovskite crystals fabricated on SiO_2 single crystal substrate. **b-d** Element mapping of 35%Br perovskite crystals. The scale bar is 10 μm. Broadband PL image of 35%Br perovskite crystals with a flat hexagonal shape on SiO_2 substrate (**e**), and the corresponding local PL evolution for different positions **f-l** on the crystal. The image is obtained by scanning with an avalanche diode detector. There are 7 points (a/b/c/d/e/f/g/h) measured here.

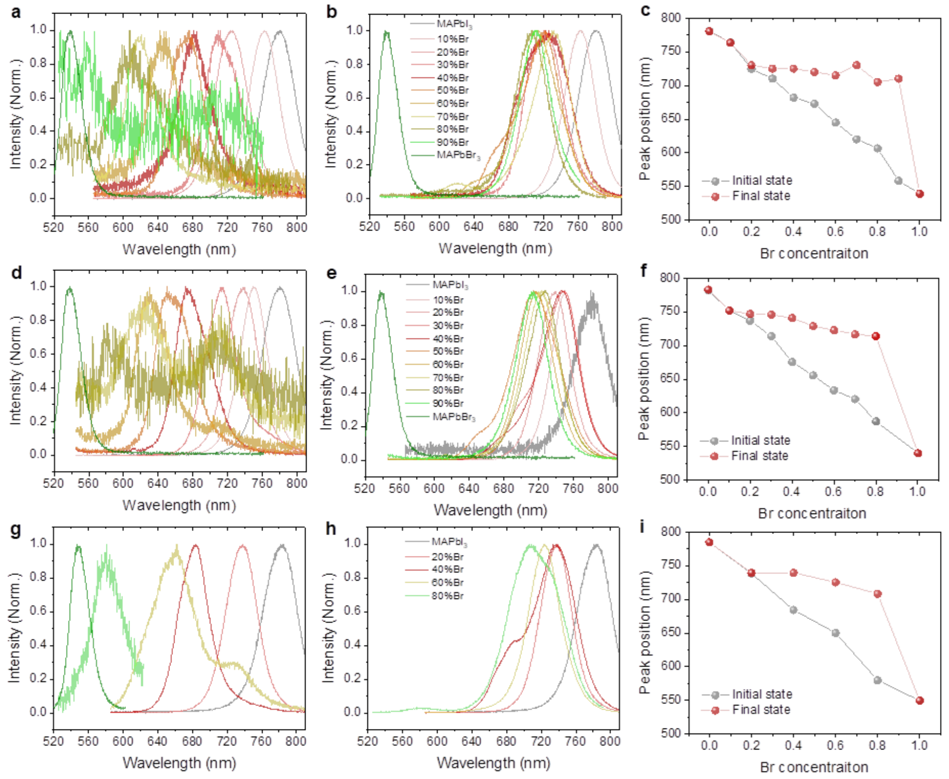

Supplementary Figure 11. PL spectra of mixed-halide perovskites spin-coated on glass substrate in the initial and final state. (a-c) PL spectra of perovskites with varying Br content, fabricated through DMF solvent and CB anti-solvent, under dark **(a)**, under illumination **(b)** and the comparison of PL peak between dark and light **(c)**. **(d-f)** PL spectra of perovskites, fabricated through GBL solvent with CB as the anti-solvent. **(g-i)** PL spectra of perovskites, fabricated through GBL solvent without using anti-solvent. A continuous-wave 490nm laser with ~100 mW cm^{-2} power is used here. The initial PL spectra were collected immediately after the laser turns on while the final PL spectra were collected after around 30 seconds' continuous illumination.

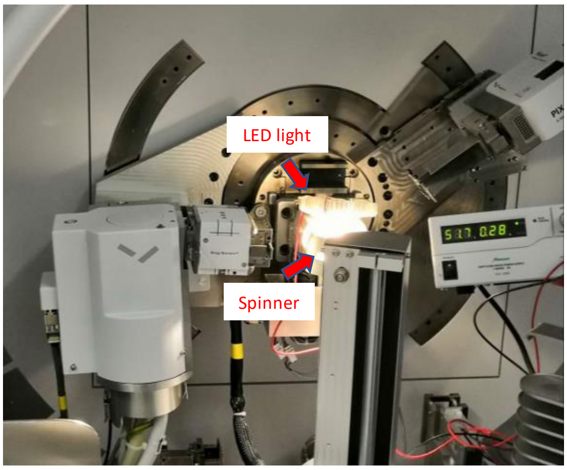

Supplementary Figure 12. The setup for *in-situ* XRD measurement under illumination. A home-made mini-LED is mounted above the spinner, which does not affect collecting the signal. The intensity of LED light is tuned by current flow. Here 0.3 A current corresponds to ~100 mW cm^{-2} intensity for 5 cm distance.

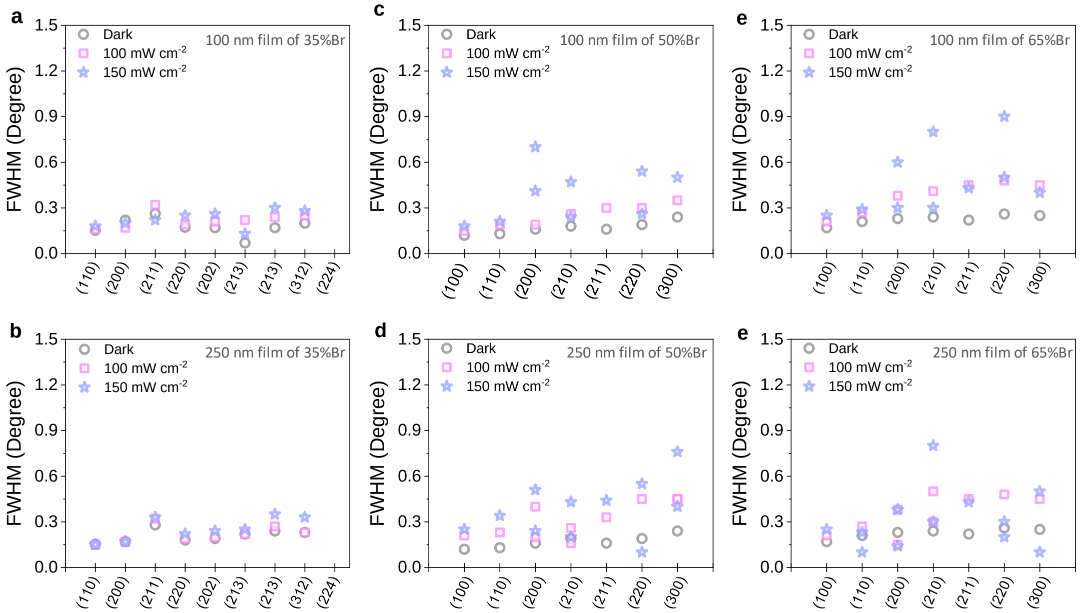

Supplementary Figure 13. a The broadening of XRD patterns under illumination for 100 nm-thick 35%Br mixed-halide perovskites. **b** The broadening of XRD patterns under illumination for 250 nm-thick 35%Br mixed-halide perovskites. **c-d** The broadening of XRD patterns under illumination for 100 nm-thick and 250 nm-thick 50%Br mixed-halide perovskites, respectively. **e-f** The broadening of XRD patterns under illumination for 100 nm-thick and 250 nm-thick 65%Br mixed-halide perovskites, respectively.

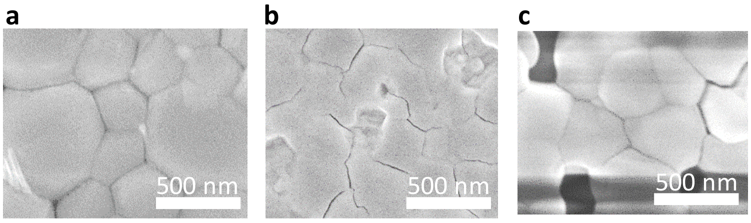

Supplementary Figure 14. SEM images of 35%Br **(a)**, 65%Br **(b)** perovskites and 65%Br perovskites with excess 20% KI **(c).**

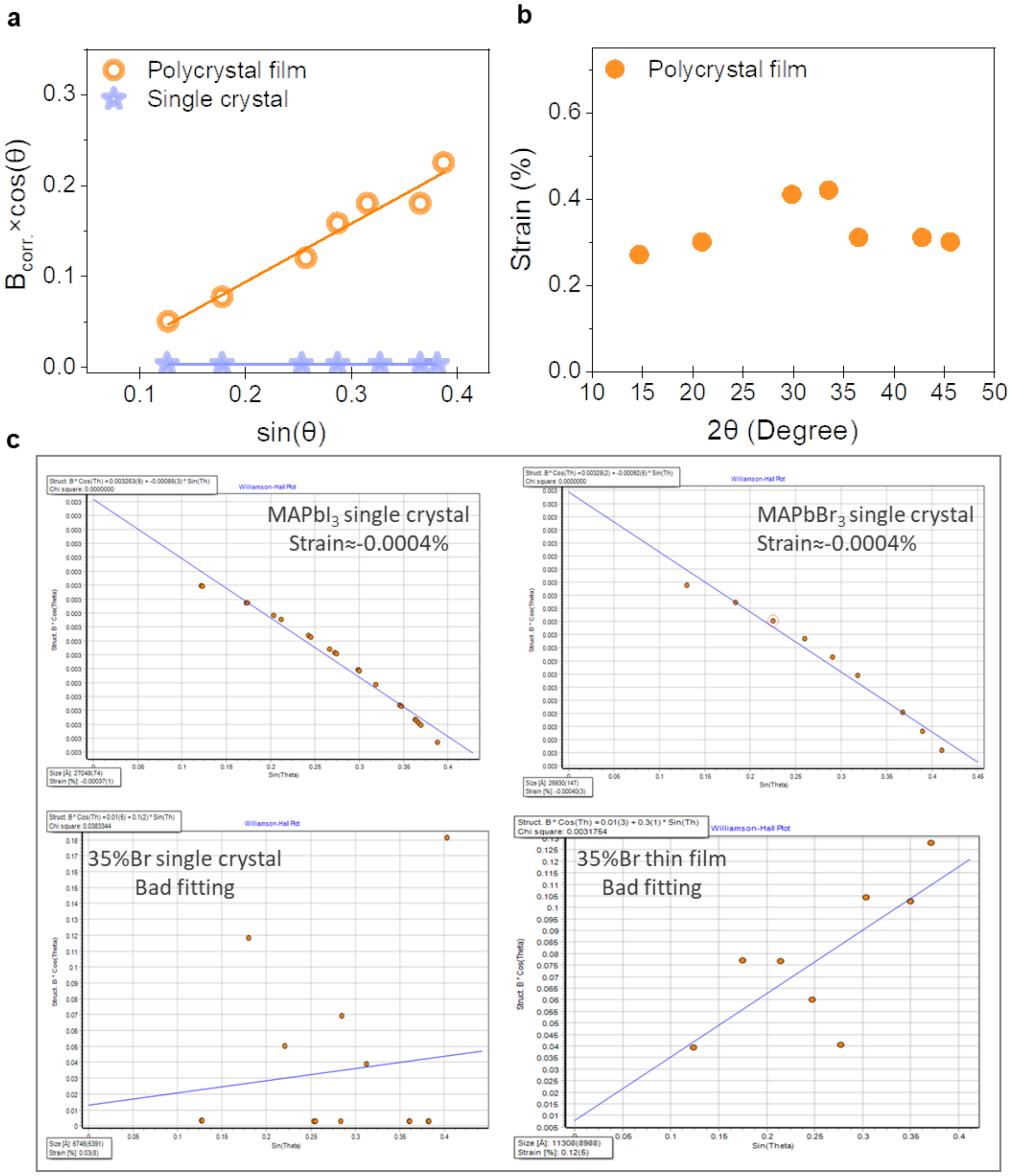

Supplementary Figure 15. Strain analysis in mixed-halide perovskites. a Williamson-Hall plot of perovskite thin film of 65% fabricated through antisolvent method, in which $B_{corr.}$ represents the broadening corrected by instrumental broadening using X'Pert HighScore Plus software. **b** Relative strain of perovskite film to the single crystal of 65% using the formula (1). **c** Williamson-Hall plot of single-crystal of $MAPbI_3$, $MAPbBr_3$, 35%Br and polycrystal thin-film 35%Br. The strain value is presented in the figure. The bad fitting of Williamson-Hall plot for 35%Br is caused by tetragonal phase-related broadening, for which we use 65%Br perovskite to compare the strain between single crystal and polycrystal.

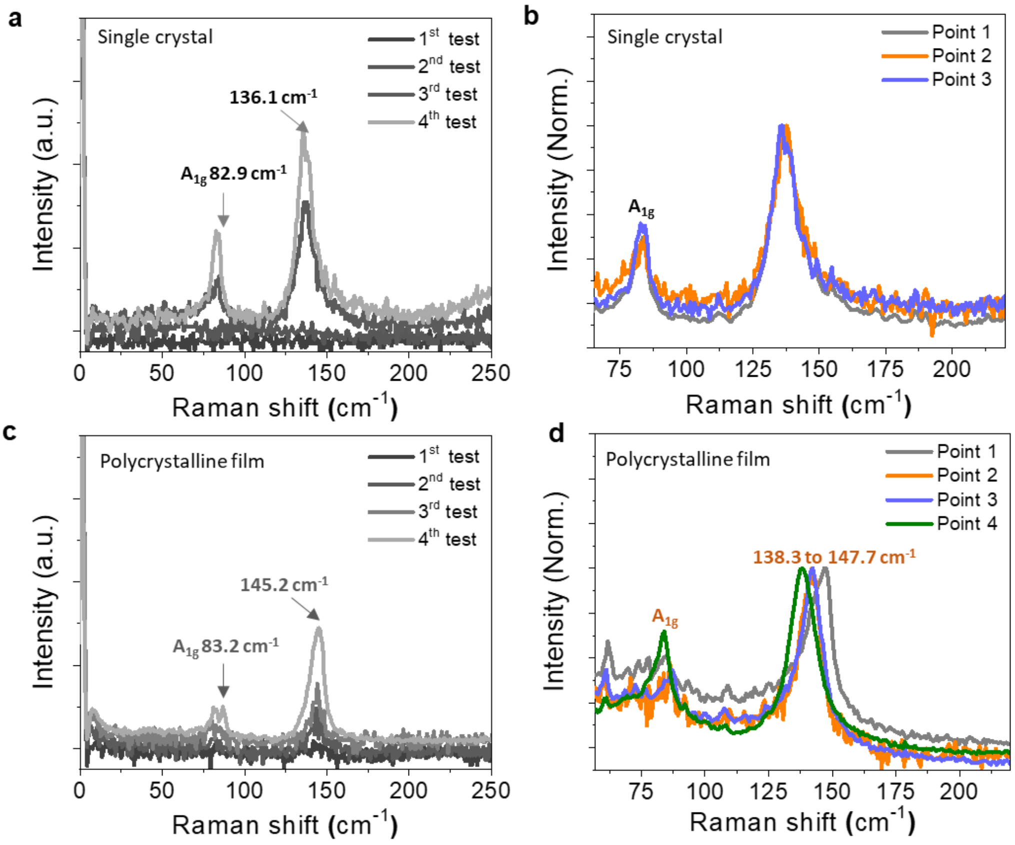

Supplementary Figure 16. **a** Raman spectra for the decomposition process of $MAPbI_3$ single crystal during Raman measurement at 20°C. **b** Raman spectra at different positions (1-3) in single-crystal $MAPbI_3$ after 20s laser illumination at 20°C. **c** Raman spectra for the decomposition process of $MAPbI_3$ polycrystalline film. **d** Raman spectra at different positions (1-4) in $MAPbI_3$ polycrystalline film after 20s laser illumination at 20°C. The integration time is 10 s for each test and the time interval in between is 0.1 s. Note that perovskite is Raman-inactive at room temperature; thus we use the laser-induced PbI_x to make the comparison. 488 nm CW laser is used here

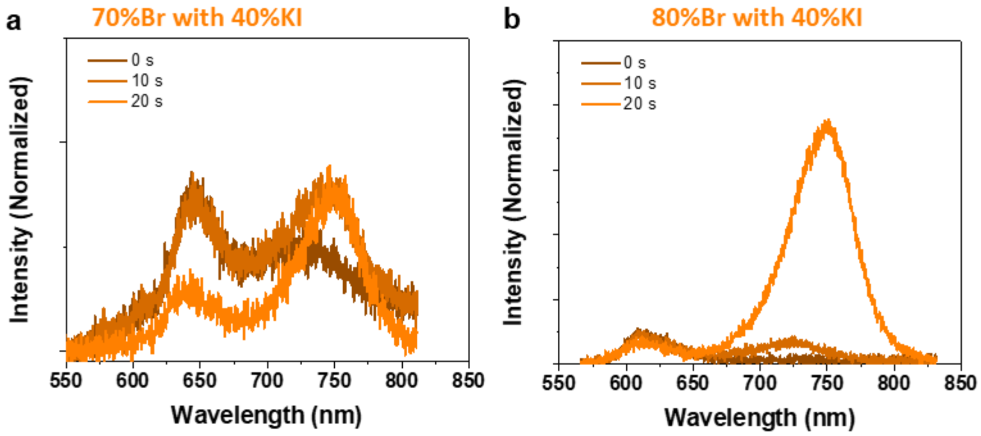

Supplementary Figure 17. PL evolution of 65%Br **(a)** and 80%Br **(b)** perovskites fabricated through the perovskite-precursor solution with excess 40%KI additives. The perovskite solution and KI solution were prepared separately with 1 M concentration and then mix 0.4 mL KI solution with 1 mL perovskite solution. 490 laser with ~100 mW cm^{-2} power is used here. Integration time for each measurement is 1 second.

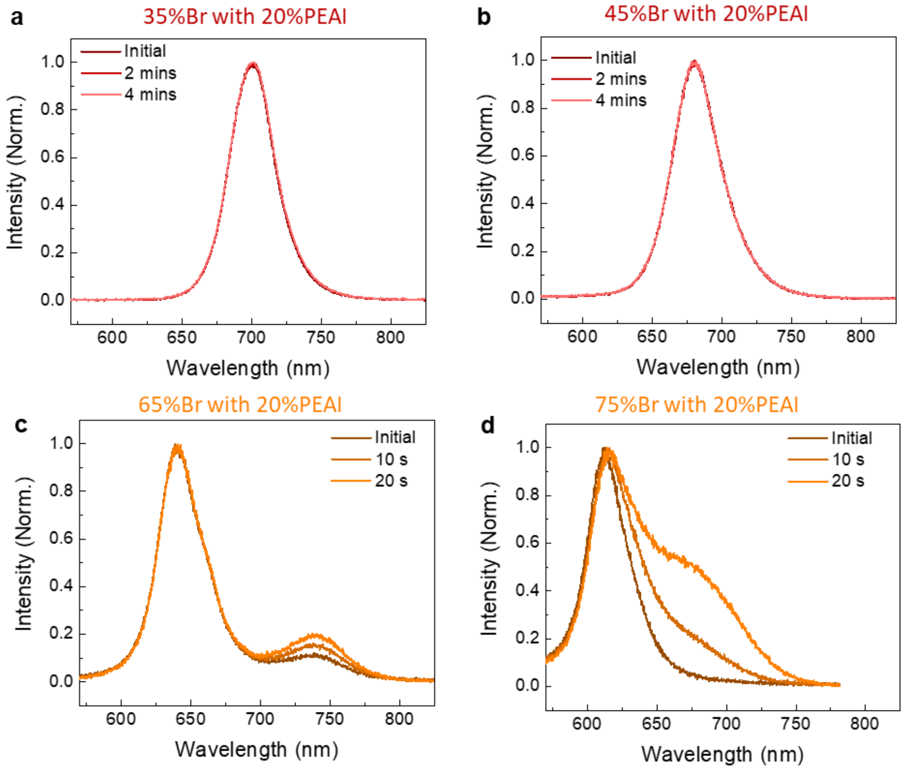

Supplementary Figure 18. PL spectra of mixed-halide perovskites synthesized from precursor solution containing excess 20 mol.% PEAI additives for 35%Br **(a)**, 45%Br **(b)**, 65%Br **(c)**, 75%Br **(d).** The perovskite solution and PEAI solution were prepared separately with 1 M concentration and then mix 0.2 mL PEAI solution with 1 mL perovskite solution. 490 laser with ~100 mW cm^{-2} power is used here. Integration time for each measurement is 1 second.

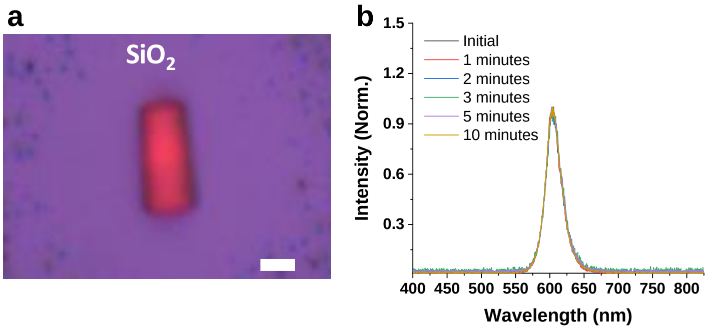

Supplementary Figure 19. Optical image **(a)** and PL evolution **(b)** of $CsPb(I_{0.35}Br_{0.65})_3$ crystals on SiO_2 substrate. 532 nm laser with 0.1 μW power is used here. The integration time for each measurement is 5 seconds.

Supplementary references:

1. Neumann, C. *et al.* Raman spectroscopy as probe of nanometre-scale strain variations in graphene. *Nature communications* **6**, 8429 (2015).
2. Veber, A., Cicconi, M.R., Reinfelder, H. & de Ligny, D. Combined Differential scanning calorimetry, Raman and Brillouin spectroscopies: A multiscale approach for materials investigation. *Anal Chim Acta* **998**, 37-44 (2018).
3. Shkir, M. *et al.* A facile synthesis of Au-nanoparticles decorated PbI2 single crystalline nanosheets for optoelectronic device applications. *Scientific reports* **8**, 13806 (2018).
4. Chen, Q. *et al.* Multiple-Stage Structure Transformation of Organic-Inorganic Hybrid Perovskite $CH_3NH_3PbI_3$. *Physical Review X* **6**, 031042 (2016).
5. Jones, T.W. *et al.* Lattice strain causes non-radiative losses in halide perovskites. *Energy & Environmental Science* **12**, 596-606 (2019).
6. Sarthak Jariwala *et al.* Local Crystal Misorientation Influences Non-Radiative Recombination. *Joule* **3**, 3048-3060 (2019).
