# Supplementary material for: Strain-activated light-induced halide segregation in mixed-halide perovskite solids
Source: Nat Commun. 2020 Dec 10;11:6328. doi: 10.1038/s41467-020-20066-7 (PMC7730187; doi:10.1038/s41467-020-20066-7)
Supplement: Supplementary file 2 — Peer Review File [file 41467_2020_20066_MOESM2_ESM.docx]

Peer Review Information

**Journal:** Nature Communications

**Manuscript title:** Strain-activated light-induced halide segregation in mixed-halide perovskite solids

**Corresponding author name(s):** Dr Yicheng Zhao

**Editorial notes:**

**none**

**Reviewer comments & decisions:**

**Reviewer comments, first version:**

Reviewer #1 (Remarks to the Author):

In this manuscript, the influence of mechanical stress on the established phenomenon of light-induced halogen segregation (LHS) in hybrid perovskite layers is carefully analyzed and discussed. The group of materials is highly relevant, in particular in research about alternative photovoltaic devices. Mobility of ions in these materials has been shown earlier to lead to both, instabilities of operation (hysteresis) and chemical instabilities of interfaces and devices. To elucidate driving forces for these processes and outline strategies to circumvent the problem as provided in this manuscript, therefore, is of high scientific interest and the work is certainly worth publishing at good visibility as provided in Nature Communications. The following comments may deserve the authors´ attention in a revision of the paper to further increase its impact:
1. (abstract) The last sentence of the abstract indicates an application of the materials as strain-gauge materials has been established in this work. Since this sentence refers to no more than an expectation at the end of the discussion, it should be omitted from the abstract.
2. (p.3 1st para) A clear correlation of LHS stated with the bands in PL observed would be helpful. It would be important to know whether or not the observed changes are reversible upon stress release. (l.95) It is hard to infer the authors assignment of signals seen in Fig. 1d: do they assign the 600 nm emission to Br0.65 and the signal at 700 nm to the segregated product (~Br0.35)? Reversible changes in XRD of Br0.65 (Fig. 3c) seem to contradict such assignment. Or do they consider the XRD to be that of the segregation product? This should be clarified.
3. (Fig. 2) To conclude the presence of strain from the shape of deposits appears reasonable but rather weak. Do the authors have independent proof? Well-defined strain can only be possible if a well-defined relative orientation of perovskite crystals and substrate exists. Is this the case?
4. (Fig. 3b,d) The symbols chosen do not allow a clear reading of the figure. It might help to use open symbols or semitransparent symbols.
5. It is not clear to this reviewer how the authors accommodate in their model apparently irreversible changes observed in PL under illumination (Fig. 4) at widely absent or reversible changes in XRD. A more explicit discussion would help.
6. (p.9 1st para) It should be pointed out that the presence of strain cannot be considered the only possible origin of peak broadening in XRD. The presence of structural defects and the size of uniformly scattering domains (effective crystal size according to Scherrer´s equation) can have similar consequences. Did the authors also apply this analysis to the data in Fig. 3a ?
7. (p9 2nd para) Raman data for MAPbI3 do not assist the discussion of the present changes. Data for Br0.35 would be helpful, but those for MAPbI3 can be omitted.
8. (p.11) While the passivation of LHS by KI or PEAI is impressive, it may have to be discussed in more detail. A grain size detected in SEM does not necessarily reflect the size of ordered domains effective in XRD. Less defects in the crystal lattice could also lead to more narrow XRD peaks. Strain-release, therefore, may of course play a role but at this point it cannot be considered proven.
9. (p12 l.264) Since iodide and bromide do not form a separate phase, it seems more accurate to speak of a statistical distribution of the two within the perovskite crystals rather than speaking of an alloy.


Reviewer #2 (Remarks to the Author):

The authors studied the connection between mechanical strains and halide segregation in mixed-halide perovskites. Light induced halide segregation has been recognized a problem for the usefulness of bandgap tuning in mixed-halide perovskites. The authors, based on photoluminescence and x-ray diffraction data, found that the halide segregation can be either facilitated or inhibited by applying external mechanical strains to single crystals or manipulating internal strains via surface modification.

While the subject of this work is certainly an important one for the development of perovskite-based optoelectronics, the findings do not necessarily advance the fundamental understandings of the light-induced halide segregation. The connection between strains and halide segregation the authors trying to elaborate was mostly phenomenological. There is not much discussion regarding the mechanisms of such connection thermodynamically or kinetically. On the other hand, this result does not present a potential solution to the halide segregation either for practical applications. Although in certain cases (e.g. excessive KI) the segregation seemed to be reduced, it was only applicable in some range of the Br content. In fact, there is a lack of investigation into the different behaviors of Br-rich vs. I-rich mixed halide perovskites.

An additional comment is about the style of writing. The manuscript was organized with a loose structure between the sections. For instance, the discussion was shifted from single crystal, to thin films, and further to polycrystalline films without a tight logic. For another example, the authors presented data based on data collected from many different compositions (Br content) and under the illumination of different wavelengths. While I am sure it is not the consequence of cherry-picking, it makes the argument hard to follow. Therefore, I’d strongly suggested the authors to recollect the data with more consistency. In addition, sufficient experimental details should be provided, especially the ones critical to the phenomena of interest such as the power density of optical illumination for this study.

In summary, the motivation of work is sound, but the design of the experiments and the interpretation of the data were not sufficiently rigorous. The results remained preliminary. The conclusions the authors attempted to reach were not quite convincing or insightful. I would not recommend this manuscript for publication in Nature Communications at the moment.


Reviewer #3 (Remarks to the Author):

Zhao et al have presented a very interesting manuscript on photo- and pressure-induced halide-demixing in single crystalline perovskite. The subject is very topical and should be of great interest to a wide readership, making it interesting for publication in a journal such as Nature Communications. Conceptually the study is very exciting but there are a number of issues (see comments) which would need to be addressed in a major revisions before this work should be accepted for publication. Unfortunately the paper has all the features of something that was put together in a rush with some lack of care. This made the reviewing process a somewhat time-consuming and frustrating experience. The phenomena described are quite complex and I don’t think the authors have made enough effort to present their findings in an easily comprehensible way.

1) Use of the term ‘light-induced halide segregation’ (LHS): in the manuscript the term LHS is also used to describe purely strain (and not photo!) induced halide segregation. This is very confusing. The authors need to be much more careful about distinguishing LHS from purely strain-activated halide segregation (SHS) which occurs even in the dark. Then again there are cases of LHS under the presence of strain. 
2) Abstract: The authors draw a black and white picture claiming that crystals with <50% Br behave intrinsically different to crystals with >50% Br. This is not supported by the data. Figure S8 shows significant LHS in absence of strain in films while for single crystals no composition between 35% and 65% were studied, to indicate that 2 crystals with 47% and 53% Br are expected to behave differently.
3) Semantics: use of the term ‘Br ratio’. While there is no such thing as a ‘Br ratio’ everybody would assume that what is referred to is the ‘Br to I ratio’. Then again the percentages provided are strictly speaking not ‘ratios’. In the ‘65% Br ratio’ sample the Br to I ratio is about 2:1.
4) Halide composition: In the paper it is assumed that the halide compositions in the crystal is the same as in the crystal growth solution. This is unfortunately somewhat common in the field. For microcrystalline films fabricated via an antisolvent strategy this can be accepted as a good estimate as the precipitation process leading to the formation of the film can be thought of as a non-discriminative process. In this sense this approximation appears acceptable, especially when this is discussed in the text. For single crystal growth we need to be concerned that the growth process is much more discriminative and that the compositions between solution and crystal varies quite significantly. The authors should attempt to analyse the composition in their single crystals.
5) Figure 1: practically all data presented in this manuscript shows increases in fluorescence intensity under continuous illumination (light soaking effect), which is common, except for the data shown in figure 1e, where the fluorescence intensity drops down to about 20% of the original value over the first 4 s of illumination. The authors need to comment on this.
6) Figure annotations: most figure annotations are inappropriate. Example: Figure 1: ‘External-pressure-induced halide segregation’. It really is fluorescence spectroscopy data and a diagram showing the experimental set up. Also there are no comments about what the times indicate. Seconds after illumination started? Seconds after pressure was applied? Figure 2: ‘Internal-strain-induced halide segregation’ I can see light microscopic images (technique is described nowhere), an SEM image and Hypospectral false colour fluorescence images of emitted light, …
7) The authors should be much more thorough when it comes to providing information about the experimental conditions. Illumination light source (steady state/pulsed), imaging technique (wide field versus confocal), excitation intensity etc. Figure 2 images don’t provide information about the length of the scale bars or any scale bar at all.
8) Formula on page 8 should be provided in modified form again (B * cos teta = …)
9) Figure 5b: the text in the figure says 65% Br and 75% Br , while the figure annotation specifies 35% Br and 50% Br
10) Figure 5c: In the manuscript the authors specify that MAPbI3 is not Raman active and that peaks that appear after continuous illumination with a focused laser originate from the decomposition product PbI2. In the figure the data is described as “Raman spectra of single crystal MAPbI3” which appears wrong. I am completely stuck for ideas what this data is supposed to tell us about LHS in mixed halide perovskites and why it was added to the manuscript. 
11) References: Again the references indicate a certain degree of carelessness, providing only the first authors for almost all papers, reference 3 lacks volume and page numbers, reference 8 misses the et al., no subscripts for perovskite formulas, throughout ref 34 missing capitalization of journal name, ‘(camb)’ in ref 31, ref 39 no page and volume number, 39, journal name not abbreviated, ref 44 no subscripts and missing space
12) SI: some artefacts due to pdf creation (mainly units disappearing and being replaced with empty squares (methods, fig S3)
13) Figures 1d and S3b show measurements under conditions that should result in similar behaviour (as indicated in the main text). They present Br-rich single crystals which are illuminated with 490 nm light (intensity unknown in both cases) in absence of externally applied strain. There are however differences in Br composition and sample temperature. The data in fig1d shows no demixing at all in the initial spectrum while S3b shows significant demixing already in the ‘initial’ spectrum. After 4 seconds is it striking to see that the sample in 1d has no apparent fluorescence left originating from the mixed phase while the spectra in S3b show comparable peak height for the mixed and demixed signal. The ratio of original peak height (600 nm) to final peak height (720 nm) is about 50:1 for 1d and close to 1:1 for S3b. Is the ‘initial’ spectrum shown in S3b really ‘initial’? This needs discussion and/or experimental verification.
14) Fig S12: ‘PL spectra…. under dark’. Unfortunately this is experimentally impossible. Needs clarification what the light source/excitation intensity, duration (pulse, steady state) for the PL measurement is. Please clarify. The ‘490 laser’ (light bias illumination source I assume), is it steady state or pulsed, what is the frequency and pulse width?

**Author rebuttal, first version:**

**Reviewer 1**

In this manuscript, the influence of mechanical stress on the established phenomenon of light-induced halogen segregation (LHS) in hybrid perovskite layers is carefully analyzed and discussed. The group of materials is highly relevant, in particular in research about alternative photovoltaic devices. Mobility of ions in these materials has been shown earlier to lead to both, instabilities of operation (hysteresis) and chemical instabilities of interfaces and devices. To elucidate driving forces for these processes and outline strategies to circumvent the problem as provided in this manuscript, therefore, is of high scientific interest and the work is certainly worth publishing at good visibility as provided in Nature Communications. The following comments may deserve the authors´ attention in a revision of the paper to further increase its impact.

**Response:**

First, we thank the reviewer for the appraisal of our work, and particularly appreciate his/her valuable time to give these helpful remarks which have guided us to improving this work.

1. (abstract) The last sentence of the abstract indicates an application of the materials as strain-gauge materials has been established in this work. Since this sentence refers to no more than an expectation at the end of the discussion, it should be omitted from the abstract.

**Response:**

We thank the reviewer’s advice and we deleted this sentence in our revised abstract.

2. (p.3 1st para) A clear correlation of LHS stated with the bands in PL observed would be helpful. It would be important to know whether or not the observed changes are reversible upon stress release. (l.95) It is hard to infer the authors assignment of signals seen in Fig. 1d: do they assign the 600 nm emission to Br0.65 and the signal at 700 nm to the segregated product (~Br0.35)? Reversible changes in XRD of Br0.65 (Fig. 3c) seem to contradict such assignment. Or do they consider the XRD to be that of the segregation product? This should be clarified.

**Response:**

We thank the referee’s concern about the reversibility of strain-activated LHS, which prompted us to return to the lab and check it again. For 35%Br single crystal, after we released the stress **without turning off the laser**, there is still PL splitting; when we turned off the laser, the PL splitting gradually disappears (**Fig. R1**). These results indicate that photons could stabilize the halide-segregated state even in a metastable state.

Yes, the emission centered at ~605 nm is ascribed to the 65%Br single crystal at its initial state. We observed that most of the 65%Br single crystals have a very low PL intensity initially. This PL peak for 65%Br is close to previous reports and its low PL intensity for this composition is also noted in other reports ^1^.

For the reversibility, there is actually no contradiction because it depends on **whether the illumination is on or off**. PL and XRD show the same reversibility when light is turned off. We supplemented the relevant information on Page 3.

*‘A reversible PL change is observed after the laser and external pressure are removed (Supplementary Figure 4).’*

**Figure R1 a** The PL spectra of 35%Br single crystal with halide segregation under continuous illumination (gray line) and after 2 minutes’ dark recovery (pink line). **b** The PL spectra of 65%Br single crystal with halide segregation under continuous illumination (gray line) and after 2 minutes’ dark recovery (pink line).

3. (Fig. 2) To conclude the presence of strain from the shape of deposits appears reasonable but rather weak. Do the authors have independent proof? Well-defined strain can only be possible if a well-defined relative orientation of perovskite crystals and substrate exists. Is this the case?

**Response:**

We thank the reviewer for this insightful suggestion. We believe the supplemented data could provide independent proof for the related strain.

We first tried to apply electron backscatter diffraction (EBSD) to prove the strain in perovskite by analyzing the distortion and broadening of Kikuchi patterns. Here we use an FEI Helios NanoLab 660 DualBeam FIB instrument equipped with an Oxford Symmetry Detector. We tried to adapt the beam settings reported in the literature^2^. However the pronounced topology of the perovskite flowers, in combination with the beam sensitivity of the perovskite only allows us to obtain EBSD patterns without fine structures for the two perovskite islands shown in **Fig**. R2c top and bottom, which disables us to analyze the local strain from the fine structures (distortion and broadening). But fortunately, these data enable us to derive the relative orientation between perovskite and the single-crystal substrate.

Compared with EBSD analysis of the perovskite single crystal, the on-CaF_2_ perovskite also shows a single-crystalline character with <110> orientation close to z-axis and <001> orientation close to y-axis, according to the pole figure (**Fig. R2d**). The EBSD patterns were integrated from 10 adjacent point measurements and successfully fitted by the Aztec software, leading to the aforementioned orientations. The derived orientation in the perovskite flowers is always close to <110> in z-axis (note the electron image is inclined by 70°). The best imaging conditions for EBSD analysis of the perovskites with respect to our equipment were found to be around 15 kV acceleration voltage and beam currents around 1 nA.

We move on to detect the substrate (CaF_2_), showing <111> orientation close to z-axis and <110> orientation close to y-axis from the related pole figures (**Fig. R2**). Combined with the lattice constant (Pb-I: 0.31 nm; Ca-F: 0.24 nm), we showed the top view and the side view of the lattice-mismatched interface if we assume a well-defined interface. We reason that such a large lattice mismatch needs crystal deformation to reduce the interfacial mismatch, which could lead to strain inhomogeneity in the crystal. The relevant revision is added to Page 5:

We also feel sorry for the previous mislabeled SrF_2_ (~~SrF~~_~~2~~_ CaF_2_) on Page 5-6 and Page 24-25:

*‘PL difference observed in crystals grown on different substrates, as well as the PL inhomogeneity in one single crystal is attributed to the varying strain distribution. Electron backscatter diffraction (EBSD) is used to verify the strain by examining the relative orientation between perovskite and CaF2 substrate, as shown in Fig. 2c-e. The perovskite flowers show a single-crystalline character with <110> orientation close to z-axis and the <001> orientation close to the y-axis, according to the related pole figure for three different positions on the crystal (Fig. 2d & Supplementary Figure 10). CaF2 shows <111> orientation close to z-axis and <110> orientation close to y-axis. Combined with the lattice constant, the side view of the lattice-mismatched interface with well-defined relative orientation is depicted in Figure 2e. We reason that interfacial energy minimization is accompanied by the crystal deformation to reduce the interfacial mismatch, which could lead to strain inhomogeneity in the crystal. The complete pole figure and analysis can be found in Supplementary Figure 10 and Method.’*

**Figure 2. Hyperspectral and EBSD characterization for 35%Br crystals on CaF_2_ substrate.** **a** Wide-field optical images of 35%Br perovskite crystals fabricated on ~~Sr~~CaF_2_ single-crystal substrates *via* drop-casting method. The images were obtained under 50× object lens in reflection mode. **b** Wide-field hyperspectral images of perovskite crystals fabricated on ~~Sr~~CaF_2_ single-crystal substrates. 532 nm diode continuous-wave laser is used to excite the sample with approx. 100 mW cm^-2^ intensity. This image is constructed by superposing three narrow-band PL intensity mapping at 680 nm, 710 nm and 740 nm (5 nm bandwidth). The PL mapping at 680 nm, 710 nm and 740 nm are marked as blue, green and red color, respectively. The initial PL peak is located at around 700 nm and red-shift to 740nm after halide segregation. **c** Electron backscatter image (70° tilted view) for EBSD analysis on perovskite crystals of 35%Br fabricated on CaF_2_ substrate. Three different positions are characterized, marked by 1/2/3. **d** Pole figures of the substrate CaF_2_ and 35%Br perovskite derived from Kikuchi patterns of EBSD. Scale bar: 10 μm. **e** Side-view crystal structures of CaF_2_/35%Br perovskite oriented at <111>/<110> in z-axis and <110>/<001> in y-axis, respectively. Scale bar: 10 μm.

**Figure R3 a** SEM micrograph (70° tilted view) of free-standing single crystal 35%Br perovskite with indicated region for subsequent EBSD analysis. **b** Kikuchi pattern of 35%Br single crystal as derived . **c** Kikuchi pattern of on-CaF_2_ 35%Br single crystal. **d** Pole figure of on-CaF_2_35%Br crystal. Note in Figure d: from the Kikuchi pattern, it is almost impossible to distinguish between (110) and (002) lattice planes due to a high similarity between the patterns of the two planes, which is an intrinsic and recognized character for tetragonal perovskite phase. **e** Pole figure of the CaF_2_ substrate. **f** Top-view crystal structure of perovskite and the substrate CaF_2_. Top left cuboid represents the unit-cell orientation of tetragonal perovskite. Bottom left cubic represents the unit-cell orientation of cubic structure CaF_2_. The sample is 35%Br perovskite, and the CIF file of MAPbI_3_ is used here considering the same crystal structure.

4. (Fig. 3b,d) The symbols chosen do not allow a clear reading of the figure. It might help to use open symbols or semitransparent symbols.

**Response:**

We thank the reviewer for this warm-hearted suggestion and we made the revision in **Figure** 3.

5. It is not clear to this reviewer how the authors accommodate in their model apparently irreversible changes observed in PL under illumination (Fig. 4) at widely absent or reversible changes in XRD. A more explicit discussion would help.

**Response:**

We feel sorry for not clarifying that local PL characterization (**Fig**. 4) was implemented under **continuous laser illumination**, whereas the ‘Dark recovery’ in XRD (**Fig**. 3) denotes the sample after **approx. 3 minutes stored under dark**. As discussed in **Response 2:** PL change is also reversible once the samples were stored under dark for a while. The reversibility of LHS has also been discussed by Richard H. Friend, Michael D. McGehee, Masaru Kuno *et al.* ^1,3,4^. In our revised manuscript, we supplemented the measurement condition and the related discussion on the reversible change. On Page 6:

*‘All the peaks recovered to their original positions when the samples were stored in dark for approx. 2 minutes. The reversibility of XRD patterns is consistent with the reversible PL change* ***(Fig. S4****) ^3,5^.’*

*‘The XRD of dark recovery indicates the sample was stored under dark for around 3 minutes after the LED light was turned off.’*

6. (p.9 1st para) It should be pointed out that the presence of strain cannot be considered the only possible origin of peak broadening in XRD. The presence of structural defects and the size of uniformly scattering domains (effective crystal size according to Scherrer´s equation) can have similar consequences. Did the authors also apply this analysis to the data in Fig. 3a ?

**Response:**

As the reviewer mentioned, the broadening in XRD is related to many factors. Here in our manuscript, we assume that the broadening mainly contains three parts: instrumental effect, size effect, and strain effect. It’s true that the structural defects like Schottky defect should also be included; however, it turns out that they do not greatly affect the XRD broadening, because the derived strain value under our assumption is close to Williamson-Hall analysis that is based on the **proportionality** between *Bcos(θ)* of *sin(θ)*, where B and *θ* indicate the peak broadening and peak position.

We appreciate the reviewer’s careful reading of our manuscript for raising the last question. In **Figure** 5, we only showed the strain analysis for 65%/75%Br samples, not 35%Br sample, because the crystal structure of 35%Br perovskite is tetragonal leading to an intrinsic XRD splitting at plane (100) (110) (111), *etc.* ^6^ The splitting is discernable for MAPbI_3_ while they are overlapped for 35%Br perovskite. This overlap-induced broadening leads to invalid Williamson-Hall analysis. Here we show the Williamson-Hall plot of **35%Br** **poly-crystal film** below and present Williamson-Hall plot of **35%Br** **single crystal** in **Fig. S15**.

**Figure R4 a** Williamson-Hall plot of perovskite poly-crystal of 35%Br; **b** Williamson-Hall plot of single-crystal 35%Br.

7. (p9 2nd para) Raman data for MAPbI3 do not assist the discussion of the present changes. Data for Br0.35 would be helpful, but those for MAPbI3 can be omitted.

**Response:**

We thank the reviewer for raising this professional and insightful question. We agree with the reviewer that the micro-Raman data for 35%Br would be more convincing than that for MAPbI_3_ film.

However, the laser source of our confocal micro-Raman is 488 nm laser, which will induce an inhomogeneous LHS in 35%Br film during Raman measurement. This inhomogeneous LHS could induce an excess local strain at Br-rich/Br-poor interfaces^4^, which would lead to misinterpretation of strain inhomogeneity due to halide segregation for 35%Br sample.

In our humble opinion, the local strain in MAPbI_3_ polycrystalline film should be very similar to that in 35%Br film because of the same crystal structure and film morphology. Therefore, we present the Raman data for MAPbI_3_ to evidence the local strain inhomogeneity in perovskite film. In the revised manuscript, we added the figure of Raman variation for MAPbI_3_ polycrystalline film to **Figure** 5, on Page 11:

*‘we use confocal Raman to analyze the strain inhomogeneity in MAPbI_3_ film excited by a 488 nm laser, which gives an optical resolution of approximately 300 nm. Note that micro-Raman spectroscopy is sensitive to the local strain ^41,42^. We select MAPbI_3_ film instead of 35%Br film because the 488 nm laser induces inhomogeneous LHS in 35%Br film. LHS itself has been reported to increase the strain at the phase-segregated regions^15^, giving rise to artifacts when analyzing the strain-inhomogeneity..’*

**Figure 5. Strain analysis in mixed-halide perovskites. a** Williamson-Hall plot of perovskite thin film of 65% and 75%Br fabricated through antisolvent method, in which B_corr._ represents the broadening corrected by instrumental broadening using X’Pert HighScore Plus software. **b** Relative strain of perovskite film to the single crystal of 65% and 75%Br using the formula (1). **c** Raman spectra of the decomposition process of MAPbI_3_ film during Raman measurement at 20℃. The integration time is 10 s for each test and the time interval in between is 0.1 s. Note that there is no Raman signal from the fresh perovskite before degradation. 488 nm CW laser is used here. **d** Raman spectra at different positions (1-5) in thin-film MAPbI_3_ after 20s laser illumination at 20℃. Note that we selected MAPbI_3_ instead of 35%Br to perform Raman test because 35%Br perovskites have inhomogeneous LHS during Raman measurement under 488 nm CW laser.

Very recently, Prof. David Ginger *et al.* proved the strain inhomogeneity in perovskite film using state-of-the-art EBSD^2^. Particularly, they evidenced that the strain is mainly located at the grain boundary in perovskite thin film, which coincidently provides strong evidence for our claim.

8. (p.11) While the passivation of LHS by KI or PEAI is impressive, it may have to be discussed in more detail. A grain size detected in SEM does not necessarily reflect the size of ordered domains effective in XRD. Less defects in the crystal lattice could also lead to more narrow XRD peaks. Strain-release, therefore, may of course play a role but at this point it cannot be considered proven.

**Response:**

We thank the referee’s concern about KI/PEAI’s effects on LHS.

Regarding the size effect, we admit that SEM images indeed do not reflect the size of the ordered grain, as demonstrated by David S. Ginger *et al.* using electron backscatter diffraction ^2^. However, the size effect only contributes to approx. 0.02^o^ reduction even if we assume the size increases from 200 nm to 500 nm; this value is still lower than 0.07^o^ reduction due to KI additives.

For defects, although Samuel D. Stranks *et al.* reported the defect passivation’s effect of potassium on (Cs,FA,MA)Pb(I_0.85_Br_0.15_)_3_,^9^ we didn’t observe the same trend in the MAPbI*_x_*Br*_3-x_* system (**Fig. R6**).

In our opinion, the strain release might be the main reason for explaining the suppressed LHS. However, we **didn’t exclude the defect passivation’s effect on LHS** due to the fact that ion migration is always involved in LHS. On Page 11:

*‘The reduced broadening cannot be explained by size effect from the SEM images of perovskites with and without KI additive (Supplementary Figure 14). Even if we assume the grain size increases from 200 nm to 500 nm, it only contributes to approx. 0.02^o^ reduction, which is smaller than the total reduction of 0.07^o^. PL spectra do not strongly support the scenario of defects passivation of KI in our case of MAPbIxBr3-x system (Supplementary Figure 20). We, therefore, summarize that in addition to defects passivation26,46, strain release is also important to stabilize mixed-halide perovskites.’*

**Figure R6 a.** PL spectra of the 35%Br perovskite film without KI (**a**) and with KI (**b**), under the same condition (laser intensity and detection distance). Different color in each figure indicates the initial PL spectra from varying position in one sample.

9. (p12 l.264) Since iodide and bromide do not form a separate phase, it seems more accurate to speak of a statistical distribution of the two within the perovskite crystals rather than speaking of an alloy.

**Response:**

We thank the referee’s suggestion and we revised ‘alloy’ to ‘mix’ through the whole manuscript.

**Reviewer 2**

The authors studied the connection between mechanical strains and halide segregation in mixed-halide perovskites. Light induced halide segregation has been recognized a problem for the usefulness of bandgap tuning in mixed-halide perovskites. The authors, based on photoluminescence and x-ray diffraction data, found that the halide segregation can be either facilitated or inhibited by applying external mechanical strains to single crystals or manipulating internal strains via surface modification.

While the subject of this work is certainly an important one for the development of perovskite-based optoelectronics, the findings do not necessarily advance the fundamental understandings of the light-induced halide segregation. The connection between strains and halide segregation the authors trying to elaborate was mostly phenomenological. There is not much discussion regarding the mechanisms of such connection thermodynamically or kinetically.

On the other hand, this result does not present a potential solution to the halide segregation either for practical applications. Although in certain cases (e.g. excessive KI) the segregation seemed to be reduced, it was only applicable in some range of the Br content. In fact, there is a lack of investigation into the different behaviors of Br-rich vs. I-rich mixed halide perovskites.

An additional comment is about the style of writing. The manuscript was organized with a loose structure between the sections. For instance, the discussion was shifted from single crystal, to thin films, and further to polycrystalline films without a tight logic. For another example, the authors presented data based on data collected from many different compositions (Br content) and under the illumination of different wavelengths. While I am sure it is not the consequence of cherry-picking, it makes the argument hard to follow. Therefore, I’d strongly suggested the authors to recollect the data with more consistency. In addition, sufficient experimental details should be provided, especially the ones critical to the phenomena of interest such as the power density of optical illumination for this study.

In summary, the motivation of work is sound, but the design of the experiments and the interpretation of the data were not sufficiently rigorous. The results remained preliminary. The conclusions the authors attempted to reach were not quite convincing or insightful. I would not recommend this manuscript for publication in Nature Communications at the moment.

**Response:**

We first thank the reviewer for his/her valuable time and the relevant assessment of our work. Although the reviewer admitted the importance of this work, he/she may have some misunderstandings regarding the subject and logic of our work.

We admit that our work is mostly phenomenological without atomic-level discussion. This is because the dynamics of light-induced halide segregation (LHS) itself already involves excited-state physics, making it pretty challenging for theoretical simulation, not mention if the strain is further involved. It is absolutely valuable to figure out the mechanism from the first principles, but those kinds of work are beyond our current ability and the scope of this paper. Despite this, this work is the first report on the relationship between strain and halide segregation, which is systematically studied by both *in-situ* single-crystal experiments and local characterization on polycrystal films.

On the contrary, we actually think that we present a potential solution to the halide segregation either for practical applications, by releasing strain in polycrystalline films. As for the different suppression effects of KI on Br-rich and I-rich perovskites, we need to clarify that this is also one of our important findings, that is composition-dependent LHS: when Br content increases over approx. 50 mol.%, **an intrinsic LHS** is observed in the free-standing single crystals and on-substrate single crystals. In our original manuscript, we also demonstrate the impossibility of suppressing the LHS in perovskites with intrinsic **LHS (X>50%)**. Therefore, we are confused by the reviewer’s comment, *‘In fact, there is a lack of investigation into the different behaviors of Br-rich vs. I-rich mixed halide perovskites.’*

We thank the reviewer for this suggestion on the writing style. In the revised manuscript, we added some topic sentences and transition sentences in front of each paragraph. Meanwhile, we draw a flow chart of our line of thought below:

Actually, the idea behind is very simple that we study from single crystal to polycrystal:

In the revised manuscript, we supplemented the laser intensity. We feel sorry that we cannot unify the laser condition for all the PL characterizations in this work because some of the samples were measured by our collaborators. For example, the Shear-force micro-PL was implemented by the University of Tübingen where only 532 nm diode continuous-wave laser is equipped in their system. In most cases, we use 490 nm continuous-wave laser. Considering a similar penetration depth of 490 nm and 532 nm laser for perovskite film, a qualitative analysis of strain-activated LHS can be safely obtained.

In our own lab, we re-collected some of the data using two kinds of lasers with different power, showing that the qualitative analysis is not affected. Although the kinetics are slightly affected by the different laser, especially under different light intensity, however, we do believe this comparison study, as well as the conclusion, is reliable in our work.

**Figure R7** PL spectra of 65%Br perovskite film under (**a)** 490 nm continuous-wave laser with ~10 mW cm^-2^ intensity and (**b)** under 387 nm continuous-wave laser with ~10 mW cm^-2^ intensity. PL spectra of 55%Br perovskite film under 490 nm continuous-wave laser with **(c)** ~10 mW cm^-2^ intensity and **(d)** ~100 mW cm^-2^.

**Reviewer 3**

Zhao et al have presented a very interesting manuscript on photo- and pressure-induced halide-demixing in single crystalline perovskite. The subject is very topical and should be of great interest to a wide readership, making it interesting for publication in a journal such as Nature Communications. Conceptually the study is very exciting but there are a number of issues (see comments) which would need to be addressed in a major revisions before this work should be accepted for publication. Unfortunately the paper has all the features of something that was put together in a rush with some lack of care. This made the reviewing process a somewhat time-consuming and frustrating experience. The phenomena described are quite complex and I don’t think the authors have made enough effort to present their findings in an easily comprehensible way.

**Response:**

We thank the Reviewer for his/her valuable time for reviewing this work and this appraisal of the work. We also appreciate the remarks which have guided us in improving the work. We hope the reviewer finds that the upgraded work with these revisions now meets the demanding publication standards of Nature Communications.

1. Use of the term ‘light-induced halide segregation’ (LHS): in the manuscript the term LHS is also used to describe purely strain (and not photo!) induced halide segregation. This is very confusing. The authors need to be much more careful about distinguishing LHS from purely strain-activated halide segregation (SHS) which occurs even in the dark. Then again there are cases of LHS under the presence of strain.

**Response:**

We feel sorry for not presenting a clear description of our experiment. First, **light is always the prerequisite** for halide segregation in mixed-halide perovskites, termed light-induced halide segregation (LHS). From our observation, iodide and bromide are almost fully miscible in MAPbI_x_Br_3-x_ **under dark**, no matter strain exists or not. In the revised manuscript, we clearly distinguished strain-activated LHS from intrinsic LHS. On Page 1-2:

*‘Here we demonstrate strain-activated LHS in MAPb(I_1-x_Br_x_)_3_ for perovskites with X below approx.50%; the perovskites with X over approx.50% show intrinsic LHS.’*

*‘colored perovskite films are feasibly fabricated under dark with stable bandgap^12-15^; however, under illumination, photo-induced halide migration leads to bandgap instability, mainly due to notorious phenomenon of light-induced halide segregation (LHS) ^1,3-5,12,16-21^.’*

2. Abstract: The authors draw a black and white picture claiming that crystals with <50% Br behave intrinsically different to crystals with >50% Br. This is not supported by the data. Figure S8 shows significant LHS in absence of strain in films while for single crystals no composition between 35% and 65% were studied, to indicate that 2 crystals with 47% and 53% Br are expected to behave differently.

**Response:**

We thank the reviewer for this insightful comment. It is true that we cannot provide this black/white picture with a strict boundary at 50%Br. As the reviewer might know, the single crystal’s growth for MAPb(I_1-x_Br_x_)_3_ with continuous tuning of Bromide content is hard to realize, especially for x=~0.5. In most cases, a mixture of perovskites with different Br content is formed. We tried to obtain a single crystal with approx. 55%Br and 45%Br using inverse-temperature reactive crystallization, finding that 55%Br crystal still shows intrinsic LHS while 45%Br crystal (EDX shows ~43%Br) shows very stable PL under illumination.

In our revised manuscript, we added this data to **Figure** S4, on Page 3:

*‘To obtain the lower limit of Br content with intrinsic LHS, we also studied three other single crystals with 45%Br, 55%Br, 80%Br. Except for 45%Br, 55%Br/80%Br single crystals present an obvious intrinsic LHS within 10 seconds under 100 mW cm^-2^ illumination; meanwhile the PL changes faster with more Br content, making it hard to track the real initial PL (Supplementary Figure S5).’*

**Figure R8 a** PL spectra of the 55%Br perovskite single crystal, under 490 nm laser illumination (~100 mW cm^-2^). **b** PL spectra of the 45%Br perovskite single crystal under 490 nm laser illumination (~100 mW cm^-2^).

3. Semantics: use of the term ‘Br content’. While there is no such thing as a ‘Br content’ everybody would assume that what is referred to is the ‘Br to I ratio’. Then again the percentages provided are strictly speaking not ‘ratios’. In the ‘65% Br content’ sample the Br to I ratio is about 2:1.

**Response:**

We feel sorry for this non-native English writing. In the revised manuscript, we corrected the use of Br ratio and unified the use of Br content by defining this word, on Page 2:

*‘the mixed-halide perovskites (APb(I_1-x_Br_x_)_3_, A=CH_3_NH_3_/NH_2_CHNH_2_/Cs) have a capability of tuning bandgap from 1.5 eV to 2.3 eV with* ***different Br content (refers to X in (APb(I_1-x_Br_x_)_3_)****’*

4. Halide composition: In the paper it is assumed that the halide compositions in the crystal is the same as in the crystal growth solution. This is unfortunately somewhat common in the field. For microcrystalline films fabricated via an antisolvent strategy this can be accepted as a good estimate as the precipitation process leading to the formation of the film can be thought of as a non-discriminative process. In this sense this approximation appears acceptable, especially when this is discussed in the text. For single crystal growth we need to be concerned that the growth process is much more discriminative and that the compositions between solution and crystal varies quite significantly. The authors should attempt to analyse the composition in their single crystals.

**Response:**

We thank the reviewer for this professional comment. We took three crystals (MAPbI_3_, MAPb(I_0.65_Br_0.35_)_3_, MAPb(I_0.55_Br_0.45_)_3_) for EDX characterization to obtain the real Br content. It shows that the Br content is below 1%Br, 33.5%Br and 43.5%Br respectively, which is very close to the Br content in the precursor solution. In the revised manuscript, on Page 3:

*‘The composition of the single crystal is examined by energy dispersive X-ray spectra, showing a similar Br content to that in the precursor solution (Fig. S1).’*

**Figure R9** Composition analysis of single crystal MAPbI_3_ **(a)**, MAPb(I_0.65_Br_0.35_)_3_ **(b)**, MAPb(I_0.55_Br_0.45_)_3_ **(c)** *via* energy-dispersive X-ray spectra.

5. Figure 1: practically all data presented in this manuscript shows increases in fluorescence intensity under continuous illumination (light soaking effect), which is common, except for the data shown in figure 1e, where the fluorescence intensity drops down to about 20% of the original value over the first 4 s of illumination. The authors need to comment on this.

**Response:**

It’s true that the PL intensity decreases in this case. In our humble opinion, it might be attributed to the reduced funneling efficiency during the interfacial reconstruction process. In **Figure** 1e, the data is collected after I/Br are fully segregated under illumination, for which the ‘initial’ PL in **Figure** 1e is already very strong. Please find the flowchart below:

With external pressure applied to the crystal, the degree of halide segregation (*i.e.* interfacial reconstruction) is changed that might lead to a lower funneling efficiency at the iodide-rich/poor interface. Since this is just a conjecture, we only supplemented the related description on Page 4:

*‘meanwhile, a redshift from 700 nm to 720 nm with reduced intensity after we further apply approx. 100 MPa stress (Fig. 1e)‘*

*‘The pressure is applied to 65%Br single crystal after the LHS process finishes under illumination and the ‘Initial PL’ is collected after I/Br are fully segregated under illumination.’*

6. Figure annotations: most figure annotations are inappropriate. Example: Figure 1: ‘External-pressure-induced halide segregation’. It really is fluorescence spectroscopy data and a diagram showing the experimental set up. Also there are no comments about what the times indicate. Seconds after illumination started? Seconds after pressure was applied? Figure 2: ‘Internal-strain-induced halide segregation’ I can see light microscopic images (technique is described nowhere), an SEM image and Hypospectral false colour fluorescence images of emitted light, …

**Response:**

We thank the reviewer for these corrections. In our revised manuscript, the figure annotations are corrected and supplemented as shown below:

*‘****Figure 1. Photoluminescence spectra of mixed-halide perovskites under uniaxial pressure.****’*

*‘The signal was collected immediately after we turned on the laser and ‘Initial’ indicates the first measurement. The pressure was first applied to the single crystal under dark and the PL signal was collected immediately after the laser was turned on.’*

*‘****Figure 2. Hyperspectral and SEM images for 35%Br crystals on single-crystal substrates. a-b*** *Optical* *images of 35%Br perovskite crystals fabricated on SrF_2_ and MgO single-crystal substrates via drop-casting method. The images were obtained under 50× object lens with reflection mode. Scale bar: 10 μm*.*’*

***‘Figure 6. PL spectra and XRD patterns of mixed-halide perovskites with KI/PEAI additives.’***

7. The authors should be much more thorough when it comes to providing information about the experimental conditions. Illumination light source (steady state/pulsed), imaging technique (wide field versus confocal), excitation intensity etc. Figure 2 images don’t provide information about the length of the scale bars or any scale bar at all.

**Response:**

We feel sorry for this mistake and we added the scale bar of 10 μm to Figure 2. The information of excitation power or intensity *etc.* is supplemented through the whole manuscript. Unless otherwise stated, the excitation source is a CW diode laser. The micro-Raman and micro-PL is based on confocal system while the optical image is based on wide-field system.

8. Formula on page 8 should be provided in modified form again (B * cos teta = …)

**Response:**

The modified form is adopted in our revised manuscript:

$$B cos\left( \theta\right)=\frac{0.9\lambda}{D}+4\varepsilon sin(\theta)+B_{0}sin(\theta)$$

9. Figure 5b: the text in the figure says 65% Br and 75% Br, while the figure annotation specifies 35% Br and 50% Br.

**Response:**

We feel so sorry for those typo and erratum in our original manuscript. We revised the figure caption in Figure 5b:

***‘Figure 5b*** *Relative strain of perovskite film to single crystal of 65% and 75%Br using the formula of peak broadening in the main text’*

10. Figure 5c: In the manuscript the authors specify that MAPbI3 is not Raman active and that peaks that appear after continuous illumination with a focused laser originate from the decomposition product PbI2. In the figure the data is described as “Raman spectra of single crystal MAPbI3” which appears wrong. I am completely stuck for ideas what this data is supposed to tell us about LHS in mixed halide perovskites and why it was added to the manuscript.

**Response:**

We thank the reviewer for this correction. We already revised it and we supplemented the reason why we put this result here and why we select MAPbI_3_ instead of 35%Br sample, as well as why we finally present the Raman spectra of PbI_x_ due to MAPbI_3_ decomposition.

To evidence the strain inhomogeneity and correlated it with the inhomogeneous LHS in poly-crystal film, we initially planned to detect the local Raman of perovskite film. However, detecting the local strain in perovskite needs a **cryogenic** confocal micro-Raman setup with the ability to detect low wavenumber (approx. 50 cm^-1^), because perovskite is not Raman active at room temperature ^22^.

However, we found that the decomposition product (PbI_X_) has a clear Raman signal at room temperature. We further reason that PbI_X_ can be used to indirectly reflect the local strain in perovskite film since it interacts with the surrounding perovskite matrix. It turns out that the Raman spectra of PbI_X_ have a large variation across the perovskite layer, which is used to evidence the strain inhomogeneity in the pristine perovskite film.

We clarified this question in our revised manuscript, on Page 9:

*‘Raman spectra of PbI_x_ have a large variation across the perovskite layer (between 138 cm^-1^ and 148 cm^-1^). We reason that the local strain of PbI_x_ can reflect the strain distribution in perovskite film since it interacts with the surrounding perovskite matrix^7^ (****Fig. 5d****). Our result is consistent with a recent work by T. Jones et al. and S. Jariwala et al. that demonstrates complex strain heterogeneity in mixed-halide perovskite films, particularly the local strain at grain boundaries ^23,2^.’*

***Figure 5 ‘c*** *Raman spectra of the decomposition process of MAPbI_3_ film during Raman measurement at 20℃. The integration time is 10 s for each test and the time interval in between is 0.1 s. Note that there is no Raman signal from the fresh perovskite before degradation. 488 nm CW laser is used here.* ***d*** *Raman spectra at different positions (1-5) in thin-film MAPbI_3_ after 20s laser illumination at 20℃. Note that we selected MAPbI_3_ instead of 35%Br to perform Raman test because 35%Br perovskites have inhomogeneous LHS during Raman measurement under 488 nm CW laser.*

***’***

During the revision process, we noticed that Prof. David Ginger *et al.* proved the strain inhomogeneity in perovskite film using state-of-the-art EBSD^2^. Particularly, they evidenced that the strain is mainly located at the grain boundary in perovskite thin film, which coincidently provides strong evidence for our claim.

11. References: Again the references indicate a certain degree of carelessness, providing only the first authors for almost all papers, reference 3 lacks volume and page numbers, reference 8 misses the et al., no subscripts for perovskite formulas, throughout ref 34 missing capitalization of journal name, ‘(camb)’ in ref 31, ref 39 no page and volume number, 39, journal name not abbreviated, ref 44 no subscripts and missing space

**Response:**

We thank the reviewer for these warm suggestions. We check the format of Nature Communications and normally we just need the First Author et al. for author number exceeding 5. In the revised manuscript, we have corrected the subscripts for many chemical formulas and the missing volumes. We again feel sorry for this carelessness.

12. SI: some artefacts due to pdf creation (mainly units disappearing and being replaced with empty squares (methods, fig S3)

**Response:**

It should be something wrong during the pdf conversion process and we uploaded a new PDF file.

13. Figures 1d and S3b show measurements under conditions that should result in similar behaviour (as indicated in the main text). They present Br-rich single crystals which are illuminated with 490 nm light (intensity unknown in both cases) in absence of externally applied strain. There are however differences in Br composition and sample temperature. The data in fig1d shows no demixing at all in the initial spectrum while S3b shows significant demixing already in the ‘initial’ spectrum. After 4 seconds is it striking to see that the sample in 1d has no apparent fluorescence left originating from the mixed phase while the spectra in S3b show comparable peak height for the mixed and demixed signal. The ratio of original peak height (600 nm) to final peak height (720 nm) is about 50:1 for 1d and close to 1:1 for S3b. Is the ‘initial’ spectrum shown in S3b really ‘initial’? This needs discussion and/or experimental verification.

**Response:**

For the light intensity, it is around 100 mW cm^-2^ unless otherwise stated (added to the caption).

The sample is 65%Br in **Figure** 1d while it’s 80%Br in **Figure** S3b. With more and more Br in MAPb(I_1-x_Br_x_)_3_, the dynamics of LHS becomes faster and faster. Normally, the integration time for each steady-state PL measurement is 1 second. To obtain the real initial PL for 80%Br, we need to collect the PL spectra quickly within 0.1 second after we turn on the laser (Fig. R9). We verified that there is no demixing in the initial state for 80%Br single crystal.

As the reviewer knows, the ratio of final peak to initial peak is decided by both the initial PL intensity and the funneling efficiency after halide segregation. Note that initial PL intensity is always very weak for 65%Br samples, no matter for single crystal or poly-crystal (Figure 1d/Figure S4c/Figure S6/Figure S17). The difference between the two crystals can be attributed to a different number of non-radiative defects at the surfaces for 65%Br and 80%Br crystals. In **Fig**. 1d with 65%Br, the original PL peak is so weak that it is quickly hidden by the PL emitted from I-rich clusters. In **Fig**.S5 for 80%Br single crystal, the initial PL intensity is relatively high.

**Figure R10** Under the 490 nm laser illumination (~100 mW cm^-2^), PL spectra of the 80%Br perovskite. Note that the integration time is 0.1 second for each measurement.

14. Fig S12: ‘PL spectra…. under dark’. Unfortunately, this is experimentally impossible. Needs clarification what the light source/excitation intensity, duration (pulse, steady state) for the PL measurement is. Please clarify. The ‘490 laser’ (light bias illumination source I assume), is it steady state or pulsed, what is the frequency and pulse width?

**Response:**

We feel sorry for not making it correctly and clearly, Through the whole manuscript, we use continuous-wave laser with *~100 mW cm^-2^* unless otherwise stated. In the revised manuscript, we added the related information to the figure caption, on Page 25:

*‘****Fig. S12. PL spectra of mixed-halide perovskites spin-coated on glass substrate in the initial and final state.*** ***(a-c)*** *PL spectra of perovskites with varying Br content, fabricated through DMF solvent and CB anti-solvent, under dark (a), under illumination (b) and the comparison of PL peak between dark and light (c).* ***(d-f)*** *PL spectra of perovskites, fabricated through GBL solvent with CB as the anti-solvent.* ***(g-i)*** *PL spectra of perovskites, fabricated through GBL solvent without using anti-solvent. A continuous-wave 490nm laser with ~100 mW cm^-2^ power is used here. The initial PL spectra were collected immediately after the laser turns on while the final PL spectra were collected after around 30 seconds’ continuous illumination.’*

1	Slotcavage, D. J., Karunadasa, H. I. & McGehee, M. D. Light-Induced Phase Segregation in Halide-Perovskite Absorbers. *ACS Energy Letters* **1**, 1199-1205, doi:10.1021/acsenergylett.6b00495 (2016).

2	Sarthak Jariwala *et al.* Local Crystal Misorientation Influences Non-Radiative Recombination. *Joule* **10**, 1903, doi:<https://doi.org/10.1016/j.joule.2019.11.001> (2019).

3	Brennan, M. C., Draguta, S., Kamat, P. V. & Kuno, M. Light-Induced Anion Phase Segregation in Mixed Halide Perovskites. *ACS Energy Letters* **3**, 204-213, doi:10.1021/acsenergylett.7b01151 (2017).

4	Hoke, E. T. *et al.* Reversible photo-induced trap formation in mixed-halide hybrid perovskites for photovoltaics. *Chem. Sci.* **6**, 613-617, doi:10.1039/c4sc03141e (2015).

5	Zhao, Y. *et al.* Quantification of light-enhanced ionic transport in lead iodide perovskite thin films and its solar cell applications. *Light: Science & Applications* **6**, e16243, doi:10.1038/lsa.2016.243 (2016).

6	Baikie, T. *et al.* Synthesis and crystal chemistry of the hybrid perovskite (CH3NH3)PbI3 for solid-state sensitised solar cell applications. *Journal of Materials Chemistry A* **1**, 5628, doi:10.1039/c3ta10518k (2013).

7	Neumann, C. *et al.* Raman spectroscopy as probe of nanometre-scale strain variations in graphene. *Nature communications* **6**, 8429, doi:10.1038/ncomms9429 (2015).

8	Veber, A., Cicconi, M. R., Reinfelder, H. & de Ligny, D. Combined Differential scanning calorimetry, Raman and Brillouin spectroscopies: A multiscale approach for materials investigation. *Anal Chim Acta* **998**, 37-44, doi:10.1016/j.aca.2017.09.045 (2018).

9	Abdi-Jalebi, M. *et al.* Maximizing and stabilizing luminescence from halide perovskites with potassium passivation. *Nature* **555**, 497-501, doi:10.1038/nature25989 (2018).

10	Barker, A. J. *et al.* Defect-Assisted Photoinduced Halide Segregation in Mixed-Halide Perovskite Thin Films. *ACS Energy Letters* **2**, 1416-1424, doi:10.1021/acsenergylett.7b00282 (2017).

11	Belisle, R. A. *et al.* Impact of Surfaces on Photoinduced Halide Segregation in Mixed-Halide Perovskites. *ACS Energy Letters* **3**, 2694-2700, doi:10.1021/acsenergylett.8b01562 (2018).

12	Levchuk, I. *et al.* Brightly Luminescent and Color-Tunable Formamidinium Lead Halide Perovskite FAPbX3 (X = Cl, Br, I) Colloidal Nanocrystals. *Nano letters* **17**, 2765-2770, doi:10.1021/acs.nanolett.6b04781 (2017).

13	Protesescu, L. *et al.* Nanocrystals of Cesium Lead Halide Perovskites (CsPbX(3), X = Cl, Br, and I): Novel Optoelectronic Materials Showing Bright Emission with Wide Color Gamut. *Nano letters* **15**, 3692-3696, doi:10.1021/nl5048779 (2015).

14	Noh, J. H., Im, S. H., Heo, J. H., Mandal, T. N. & Seok, S. I. Chemical management for colorful, efficient, and stable inorganic-organic hybrid nanostructured solar cells. *Nano letters* **13**, 1764-1769, doi:10.1021/nl400349b (2013).

15	Zhang, Y., Liu, Y., Li, Y., Yang, Z. & Liu, S. Perovskite CH3NH3Pb(BrxI1−x)3 single crystals with controlled composition for fine-tuned bandgap towards optimized optoelectronic applications. *Journal of Materials Chemistry C* **4**, 9172-9178, doi:10.1039/c6tc03592b (2016).

16	Hu, M., Bi, C., Yuan, Y., Bai, Y. & Huang, J. Stabilized Wide Bandgap MAPbBrxI3-xPerovskite by Enhanced Grain Size and Improved Crystallinity. *Advanced Science* **3**, 1500301, doi:10.1002/advs.201500301 (2016).

17	Bischak, C. G. *et al.* Origin of Reversible Photoinduced Phase Separation in Hybrid Perovskites. *Nano letters* **17**, 1028-1033, doi:10.1021/acs.nanolett.6b04453 (2017).

18	Draguta, S. *et al.* Rationalizing the light-induced phase separation of mixed halide organic-inorganic perovskites. *Nature communications* **8**, 200, doi:10.1038/s41467-017-00284-2 (2017).

19	Gualdron-Reyes, A. F. *et al.* Controlling the Phase Segregation in Mixed Halide Perovskites through Nanocrystal Size. *ACS Energy Lett* **4**, 54-62, doi:10.1021/acsenergylett.8b02207 (2019).

20	Mao, W. *et al.* Visualizing Phase Segregation in Mixed-Halide Perovskite Single Crystals. *Angewandte Chemie* **58**, 2893-2898, doi:10.1002/anie.201810193 (2019).

21	Wang, X. *et al.* Suppressed phase separation of mixed-halide perovskites confined in endotaxial matrices. *Nature communications* **10**, 695, doi:10.1038/s41467-019-08610-6 (2019).

22	Chen, Q. *et al.* Multiple-Stage Structure Transformation of Organic-Inorganic Hybrid Perovskite CH_3_NH_3_PbI_3_. *Physical Review X* **6**, 031042 doi:10.1103/PhysRevX.6.031042 (2016).

23	Jones, T. W. *et al.* Lattice strain causes non-radiative losses in halide perovskites. *Energy & Environmental Science* **12**, 596-606, doi:10.1039/c8ee02751j (2019).

**Reviewer comments, second version:**

Reviewer #1 (Remarks to the Author):

It is appreciated that the authors went back to look at their data in view of the comments by the reviewers and invested additional efforts to clarify the situation. However, I must say that they did not at all manage to present a comprehensive view. Rather, some additional thoughts are listed, but are not embedded in the overall argument of the paper. Therefore, I cannot recommend publication. This is a pity since the authors did valuable experiments. The discussion and presentation, however, do not reach the high standards of nature communication. In the following, I am commenting on the authors´ response to my original comment according to their numbers:

1. OK
2. A clear assignmnt of PL bands to different species is still missing. The text describes the information in the Figure 1, but does not discuss it in its assignment and meaning. Further, reversibility of the changes observed in PL is now mentioned, but not properly discussed. What kind of mechanism of (reversible) segregation has to be assumed? How can light stabilize the segregated phase? How can the back-reaction occurr in the dark? These points are not clarified, the underlying problems are not even mentioned, the facts are just described.
3. A rather demanding crystallographic analysis is now added. While this is appreciated, it must be stated that quite hand-waving arguments are used, no references are included to discuss the method an the results and the experimental procedure is decribed in a Figure legend only. The reader has no chance to evaluate the relevance of these measurements and their meaning to proof strain in the samples.
4. OK
5. (adding to 2) Indeed, these two sets of experiments now are fitting much better than in the original version. However, it is not at all clear to this reviewer, how the authors can derive the storage time in the dark from the observed intensity changes in XRD (new sentence in the legend of Figure 3).
6. In their response the authors provide additional arguments in favor of their interpretation. However, such arguments would have to be included in the discussion in the manuscript in order to provide a straightforward discussion to the reader.
7. No doubt that strain in these samples can vary locally. This reviewer, however, does not see the relevance of a detailed vibrational analysis of a chemical decomposition product (namely PbI2) in samples of MAPbI3 in order to gain insight into the local strain distribution in a different material (Br0.35) which even undergoes halide segregation. Rather than adding more and more experimental results, a conclusive discussion of the PL and structural data would have helped to support the authors´ line of argument.
8. Ok in general but an argument is missing in the text why the measured "PL spectra do not strongly support the scenario of defects passivation". It is the task of the authors to list such argument rather than a task of the reader to find it.
9. OK.

In summary, I come to the conclusion that the authors did not succeed in providing a solid set of straightforwardly arranged arguments in order to make their point.


Reviewer #3 (Remarks to the Author):

I would like to thank the authors for the extensive revision of their manuscript and the detailed response to al of the reviewers questions and comments. After this major revision I now feel that the paper should be accepted for publication in Nature Communications.
Minor comment for figure 5: consider replacing 'fresh' with 'as prepared'.

**Author rebuttal, second version:**

**Reviewer 1**

**p.s. The previous review is attached at the end**

**Comments from Reviewer 1:**

It is appreciated that the authors went back to look at their data in view of the comments by the reviewers and invested additional efforts to clarify the situation. However, I must say that they did not at all manage to present a comprehensive view. Rather, some additional thoughts are listed, but are not embedded in the overall argument of the paper. Therefore, I cannot recommend publication. This is a pity since the authors did valuable experiments. The discussion and presentation, however, do not reach the high standards of nature communication. In the following, I am commenting on the authors´ response to my original comment according to their numbers:

**Response:**

We sincerely appreciate your valuable comments which have guided us in improving this work. We feel sorry for the poor readability of our last manuscript. It failed to articulate the key findings and the argumentation with a comprehensive view and unfortunately caused some misunderstanding. After carefully polishing the whole manuscript, we think the line-of-thought in this work is now comprehensively articulated. We hope you find this brand-new version deserves your re-evaluation. Considering the revision of many details, only the main revisions are marked with red color.

The point-by-point response and **a summary of the main amendments** are listed below:

1. OK.
2. A clear assignment of PL bands to different species is still missing. The text describes the information in the Figure 1, but does not discuss it in its assignment and meaning. Further, reversibility of the changes observed in PL is now mentioned, but not properly discussed. What kind of mechanism of (reversible) segregation has to be assumed? How can light stabilize the segregated phase? How can the back-reaction occur in the dark? These points are not clarified, the underlying problems are not even mentioned, the facts are just described.

**Response:**

**Regarding the *‘its assignment and meaning’:*** We think the reviewer is asking the content of Br in the light-induced iodide-rich clusters. We supplement the content in our revised manuscript based on the PL peak.

**Page 3, Line 83:**

1

35%Br single crystal does not present LHS before applying the external stress, indicated by a stable PL at ca.704 nm, consistent with the bandgap of as-prepared MAPb(I0.65Br0.35)3

**Page 3, Line 85:**

The second PL at 740 nm is assigned to the formation of MAPb(I0.85Br0.15)3.

**Page 3, Line 94:**

The initial PL at 600 nm corresponds to the bandgap of as-prepared 65%Br while the final PL at 700 nm is attributed to the formation of _MAPb(I0.65Br0.35)3._ There might be a deviation for PL assignment due to quantum confinement effect of these light-induced nanosize clusters ^18,20.^

**Regarding the *‘What kind of mechanism of (reversible) segregation has to be assumed? How can light stabilize the segregated phase? How can the back-reaction occur in the dark?’:*** Previously, we didn’t extend our discussion to photon’s stabilization effect and the related reversibility, because we think they deserve being studied as a new topic. An in-depth discussion was considered to exceed the scope of our current study. But still, to clarify these questions and make the entire work more readable, we add a short discussion by citing the work of Prof. Naomi S. Ginsberg *et al.* They demonstrated the polaron’s effect on stabilizing the segregated phase, as well as the reversibility (Nano letters 17, 1028-1033, DOI:10.1021/acs.nanolett.6b04453 (2017)).

**Page 3, Line 97:**

The strain-activated LHS in 35%Br crystal or intrinsic LHS in 65%Br crystal is reversible only when the laser is off (**Supplementary Figure 4**). The halide-segregation metastable state is stabilized by laser illumination, which might due to a polaron effect^18^.

3. A rather demanding crystallographic analysis is now added. While this is appreciated, it must be stated that quite hand-waving arguments are used, no references are included to discuss the method an the results and the experimental procedure is described in a Figure legend only. The reader has no chance to evaluate the relevance of these measurements and their meaning to prove strain in the samples.

**Response:**

The discussion is now well integrated into this revised manuscript with a clear interpretation. The CIF files and the step-by-step derivation for modeling the interface are also attached to **Supporting Information** of this revised manuscript. The logic of this argument is also supplemented at the beginning of the discussion:

**Page 5, Line 122-139:**

Besides mechanical pressure on single crystals, strained perovskite can also be achieved by lattice-mismatched substrate^1^. Lattice mismatch at the interface with well-defined relative orientation can introduce strain into the softer material (here the perovskite)^2,3^.

2

In this study, we fabricated 35%Br crystals on four types of single-crystal substrates (Mica, SiO2, CaF2, MgO), using drop-cast method. We observed flower-shaped structures on CaF2/MgO substrates (**Fig. 2a-2c**). To clarify whether this structure consists of adjacent multi-crystals or just one highly deformed single crystal, we characterized three different regions in one flower-like structure *via* electron backscatter diffraction (EBSD)^46^, as shown in **Supplementary Figure 7-8**. EBSD can reveal the relative orientation between perovskite and CaF2 substrate. The results indicate a single-crystal character for this structure with <110> orientation close to z-axis and the <001> orientation close to the y-axis**.** Here the z-axis is defined as the direction perpendicular to the CaF2 substrate. With the lattice constant of perovskite and CaF2, we further modeled the interface and found a large lattice mismatch (~10%) between perovskite and CaF2. The detailed analysis and CIF files can be found in **Supporting Information**. This large mismatch implies that tremendous strain energy is generated during crystal growth. This strain energy can be effectively reduced by crystal deformation, which will induce heterogeneously-strained perovskites on CaF2 or MgO, leading to inhomogeneous LHS.

**Page 20, Line 618-629:**

**Supplementary Note on Figure S7-S8**

For EBSD analysis, the pronounced topology of the perovskite flowers, in combination with the beam sensitivity of the perovskite only allows us to obtain Kikuchi patterns without fine structures for the perovskite flower shown in **Supplementary Figure 8**a. These patterns disable us to analyze the local strain from the fine structures (distortion and broadening). But fortunately, these data enable us to derive the relative orientation between perovskite and the single-crystal substrate (CaF2). Crystallographic pole figures are then extracted from the Kikuchi patterns. The pole figure of perovskite shows a single-crystalline character with <110> orientation close to z-axis and <001> orientation close to y-axis. For CaF2, it shows <111> orientation close to z-axis and <110> orientation close to y-axis. Combined with the lattice constant (Pb-I: 0.3 nm, I-I: 0.6 nm; Ca-F: 0.24 nm, Ca-Ca: 0.39 nm) and CIF files, we depict the top view and the side view of this lattice-mismatched interface (**Supplementary Figure 7f & Supplementary Figure 8c**). Z-direction indicates the direction perpendicular to CaF2 substrate here. The CIF files are attached in the raw data file.

**Regarding the *‘Hand-waving arguments’*.** We feel sorry that our unclear explanation caused some misunderstanding in the last manuscript. Considering that lattice mismatch along a well-defined crystallographic orientation of the substrate can introduce strain into the softer material, here we use electron backscatter diffraction (EBSD) to verify the lattice mismatch by examining the relative orientation between perovskite and CaF2 substrate. This logic was also pointed out by the reviewer, *‘Well-defined strain can only be possible if a well-defined relative orientation of perovskite crystals and substrate exists. Is this the case?’.* Our answer is yes.

**Page 5, Line 137:**

With the lattice constant of perovskite and CaF2, we further modeled the interface and found a large lattice mismatch (~10%) between perovskite and CaF2. The detailed analysis and CIF files can be found in **Supporting Information**. This large mismatch implies that tremendous strain energy is generated during crystal growth. This strain energy can be effectively reduced by crystal deformation, which will induce heterogeneously-strained perovskites on CaF2 or MgO, leading to inhomogeneous LHS.

3

**Regarding the references:** We feel sorry for this. We had one in the response letter, which is now added to the manuscript.

**Regarding the *‘Results and the experimental procedure in figure legend only’*:** If we had only added Figure 2, that might be true; however, we also added the methods and some supporting information, so we don’t think this is a valid point. Just to state again, EBSD is nothing exotic, but rather a well-established technique also utilized in industry. In our view, the Supporting Information is exactly the right place to show the details since this is not a paper dedicated to the EBSD technique.

**Regarding the *‘No chance to evaluate the relevance of the measurements’*:** we believe that our new interpretation will be clear to the readers.

4. OK
5. (adding to 2) Indeed, these two sets of experiments now are fitting much better than in the
   original version. However, it is not at all clear to this reviewer, how the authors can derive the storage time in the dark from the observed intensity changes in XRD (new sentence in the legend of Figure 3).

**Response:**

The time duration describes the experimental procedure, not the least time needed for XRD recovery. We feel puzzled by the word *‘derive’ in “derive the storage time in the dark from the observed intensity changes in XRD”*. The waiting time is preset, not derivation.

**Page 7, Line 186:**

The XRD of ‘Dark recovery’ was measured after the LED light was off for approx. 3 minutes.

6. In their response the authors provide additional arguments in favor of their interpretation. However, such arguments would have to be included in the discussion in the manuscript in order to provide a straightforward discussion to the reader.

**Response:**

We provided the related arguments in Supporting Information (Supplementary Figure 15) last time. Here we add more discussion to clarify the situation.

**Page 10, Line 223:**

We also note that 35%Br presents singular peak broadening caused by tetragonal phase-induced broadening.

**Page 10, Line 226:**

4

With grain size D=500 nm, the size-related broadening is 0.016° for diffraction angle at 14° and strain of 0.27% is obtained by subtracting the broadening of single crystal (0.07°) and size effect (0.016°) from the total broadening (0.17°). Single crystal is referred to as strain-free reference and equivalent to instrumental broadening here. The Schottky defect is not included for XRD broadening because the derived strain value under this assumption is close to Williamson-Hall analysis.

**Page 25, Line 674:**

The bad fitting of Williamson-Hall plot for 35%Br is caused by tetragonal phase-related broadening, for which we use 65%Br perovskite to compare the strain between single crystal and polycrystal.

7. No doubt that strain in these samples can vary locally. This reviewer, however, does not see the relevance of a detailed vibrational analysis of a chemical decomposition product (namely PbI2) in samples of MAPbI3 in order to gain insight into the local strain distribution in a different material (Br0.35) which even undergoes halide segregation. Rather than adding more and more experimental results, a conclusive discussion of the PL and structural data would have helped to support the authors´ line of argument.

**Response:**

First, we do appreciate your valuable suggestions that greatly improve the argumentation of our manuscript.

We fully agree with you that confocal-Raman data for 35%Br would be most convincing and relevant to support our scenario. However, due to our setup limitation:

1. We observe that perovskite is Raman-**inactive** at 20℃, consistent with a previous report^4^. Since we didn’t find a confocal Raman system equipped with cryogenic chamber, we have to utilize the degradation product PbIX to indirectly evidence the strain inhomogeneity.
0. **Just as you mentioned**, 35%Br will undergo inhomogeneous halide segregation during Raman measurement **with 488nm laser**. This inhomogeneous LHS will cause an artifact to the local Raman data^5^. To obtain a real strain inhomogeneity in perovskite thin films, we have to turn to MAPbI3 instead of 35%Br sample.

The above limitations indeed weaken the strength of our evidence. Meanwhile, our previous discussion was complicated and distracted the reader from the mainline of this paper.

Nevertheless, their implication on structural heterogeneity can support our scenario of strain inhomogeneity in perovskite thin films. Therefore, we transfer the Raman data to **Supporting Information**, including a detailed discussion. At the same time, a conclusive discussion on local PL and structural inhomogeneity is provided in the main text.

We believe this new layout of our manuscript, combined with a clearer discussion, will make our key findings more straightforward.

**Page 8, Line 213:**

Combined with the results on single crystals, the local LHS at grain boundaries in 35%Br films is ascribed to local strain at these regions. To support our scenario of strain inhomogeneity in perovskite films, we

5

first demonstrate an averaging 0.3% strain in the thin films using Williamson-Hall plot^5-7^. This value is much larger than that in single crystals (~0.0004%) (**Supplementary Figure 15**). Compared with single crystals, local strain characterization, via confocal Raman, further implies a pronounced strain inhomogeneity in the thin films (**Supplementary Figure 16**). A detailed analysis can be found in **Supporting Information**.

8. Argument is missing in the text why the measured "PL spectra do not strongly support the scenario of defects passivation". It is the task of the authors to list such argument rather than a task of the reader to find it.

**Response:**

We thank the reviewer for pointing out this issue. This part is now integrated into this manuscript with a new figure 5.

**Page 10, Line 248:** PL spectra do not strongly support defects reduction by KI because the PL intensity is not enhanced by KI additive in our case (**Fig. 5e**). We conclude that strain release is the main mechanism for the narrowed XRD patterns, as shown in **Figure 5f**.

**Figure 5. PL spectra and XRD patterns of mixed-halide perovskites with KI/PEAI additives. a** The schematic illustration of three distinct regimes for light-induced halide segregation in mixed-halide perovskites _MAPb(I1-xBrx)3._ With more Br, _MAPb(I1-xBrx)3_ goes through from LHS-free regime (*e.g.* MAPb(I0.85Br0.15)3) to strain-activated LHS region (*e.g.* _MAPb(I0.65Br0.35)3)_ and to LHS-intrinsic region (*e.g.* MAPb(I0.35Br0.65)3). **-b-c** PL evolution for the mixed-halide perovskite films (35%Br/45%Br/80%Br) with 20 mol.% KI passivation. The excitation wavelength is 490 nm and the laser intensity is approx. 100 mW cm^-^

6

2**. d** The XRD patterns of mixed-halide perovskites with and without KI passivation. Note that the XRD patterns of samples with KI additive are aligned with the XRD of the control samples. **e** Initial PL spectra for 35%Br perovskite films without and with 20 mol.% KI, showing a similar PL intensity. The excitation wavelength is 490 nm and the laser intensity is approx. 100 mW cm^-2^. **f** The schematic of surface passivation by KI/PEAI to release the strain in perovskites.

9. OK.

In summary, I come to the conclusion that the authors did not succeed in providing a solid set of straightforwardly arranged arguments in order to make their point.

**Response:**

After our meticulous revision, this whole manuscript now possesses better argumentation, clearer figures and re-organized presentation. We hope this new version provides a straightforward and comprehensive view of our work. For your convenience, the main changes are marked with red color.

Again, thank you very much for taking your valuable time to review this paper. **Summary of the main amendments:**

1. In each figure, labels and notations are added to present photoluminescence change at different time and different conditions;
2. At the beginning of each paragraph, subtitle and motivation of the following experiment are added;
3. In Figure 2, the EBSD data is replaced by a hyperspectral image of perovskite single crystals on MgO. The detailed EBSD analysis is transferred to Supporting Information, which can help the reader not to be distracted from the mainline of this study (strain-activated halide segregation)
4. Detailed step-by-step analysis of EBSD data is added to Supporting Information, to help the reader to assess its relevance and the meaning to prove inhomogeneous strain in the deformed crystals. The original CIF files are also attached;
5. The photoluminescence summary in Table 1 is integrated into the discussion of Figure 3 to improve the readability;
6. A schematic illustration and PL spectra are added to the new Figure 5, to make the readers easily understand the conclusion of this work;
7. The confocal Raman in original Figure 5 is transferred to Supporting Information because it will distract the readers’ attention from our key findings.

7

**Previous letter - Reviewer 1**

In this manuscript, the influence of mechanical stress on the established phenomenon of light-induced halogen segregation (LHS) in hybrid perovskite layers is carefully analyzed and discussed. The group of materials is highly relevant, in particular in research about alternative photovoltaic devices. Mobility of ions in these materials has been shown earlier to lead to both, instabilities of operation (hysteresis) and chemical instabilities of interfaces and devices. To elucidate driving forces for these processes and outline strategies to circumvent the problem as provided in this manuscript, therefore, is of high scientific interest and the work is certainly worth publishing at good visibility as provided in Nature Communications. The following comments may deserve the authors´ attention in a revision of the paper to further increase its impact.

**Response:**

First, we thank the reviewer for the appraisal of our work, and particularly appreciate his/her valuable time to give these helpful remarks which have guided us to improving this work.

1. (abstract) The last sentence of the abstract indicates an application of the materials as strain-gauge materials has been established in this work. Since this sentence refers to no more than an expectation at the end of the discussion, it should be omitted from the abstract.

**Response:**

We thank the reviewer’s advice and we deleted this sentence in our revised abstract.

2. (p.3 1st para) A clear correlation of LHS stated with the bands in PL observed would be helpful. It would be important to know whether or not the observed changes are reversible upon stress release. (l.95) It is hard to infer the authors assignment of signals seen in Fig. 1d: do they assign the 600 nm emission to Br0.65 and the signal at 700 nm to the segregated product (~Br0.35)? Reversible changes in XRD of Br0.65 (Fig. 3c) seem to contradict such assignment. Or do they consider the XRD to be that of the segregation product? This should be clarified.

**Response:**

We thank the referee’s concern about the reversibility of strain-activated LHS, which prompted us to return to the lab and check it again. For 35%Br single crystal, after we released the stress **without turning off the laser**, there is still PL splitting; when we turned off the laser, the PL splitting gradually disappears (**Fig. R1**). These results indicate that photons could stabilize the halide-segregated state even in a metastable state.

8

Yes, the emission centered at ~605 nm is ascribed to the 65%Br single crystal at its initial state. We observed that most of the 65%Br single crystals have a very low PL intensity initially. This PL peak for 65%Br is close to previous reports and its low PL intensity for this composition is also noted in other reports ^8.^

For the reversibility, there is actually no contradiction because it depends on **whether the illumination is on or off**. PL and XRD show the same reversibility when light is turned off. We supplemented the relevant information on Page 3.

*‘A reversible PL change is observed after the laser and external pressure are removed (Supplementary Figure 4).’*

**a**	35% single crystal	**b**	65% single crystal

**Figure R1 a** The PL spectra of 35%Br single crystal with halide segregation under continuous illumination (gray line) and after 2 minutes’ dark recovery (pink line). **b** The PL spectra of 65%Br single crystal with halide segregation under continuous illumination (gray line) and after 2 minutes’ dark recovery (pink line).

**600 650 700 750 800 850**

**Wavelength (nm)**

**550 600 650 700 750 800**

**Wavelength (nm)**

**Intensity (a.u.)**

Before dark recovery

After dark recovery (2 minutes)

**Intensity (a.u.)**

Before dark recovery

After dark recovery (2 minutes)

3. (Fig. 2) To conclude the presence of strain from the shape of deposits appears reasonable but rather weak. Do the authors have independent proof? Well-defined strain can only be possible if a well-defined relative orientation of perovskite crystals and substrate exists. Is this the case?

**Response:**

We thank the reviewer for this insightful suggestion. We believe the supplemented data could provide independent proof for the related strain.

We first tried to apply electron backscatter diffraction (EBSD) to prove the strain in perovskite by analyzing the distortion and broadening of Kikuchi patterns. Here we use an FEI Helios NanoLab 660 DualBeam FIB instrument equipped with an Oxford Symmetry Detector. We tried to adapt the beam settings reported in the literature^9^. However the pronounced topology of the perovskite flowers, in combination with the beam sensitivity of the perovskite only allows us to obtain EBSD patterns without fine structures for the two perovskite islands shown in **Fig**. R2c top and bottom, which disables us to analyze the local strain from the fine structures

9

(distortion and broadening). But fortunately, these data enable us to derive the relative orientation between perovskite and the single-crystal substrate.

Compared with EBSD analysis of the perovskite single crystal, the on-CaF2 perovskite also shows a single-crystalline character with <110> orientation close to z-axis and <001> orientation close to y-axis, according to the pole figure (**Fig. R2d**). The EBSD patterns were integrated from 10 adjacent point measurements and successfully fitted by the Aztec software, leading to the aforementioned orientations. The derived orientation in the perovskite flowers is always close to <110> in z-axis (note the electron image is inclined by 70°). The best imaging conditions for EBSD analysis of the perovskites with respect to our equipment were found to be around 15 kV acceleration voltage and beam currents around 1 nA.

We move on to detect the substrate (CaF2), showing <111> orientation close to z-axis and <110> orientation close to y-axis from the related pole figures (**Fig. R2**). Combined with the lattice constant (Pb-I: 0.31 nm; Ca-F: 0.24 nm), we showed the top view and the side view of the lattice-mismatched interface if we assume a well-defined interface. We reason that such a large lattice mismatch needs crystal deformation to reduce the interfacial mismatch, which could lead to strain inhomogeneity in the crystal. The relevant revision is added to Page 5:

We also feel sorry for the previous mislabeled SrF2 (SrF2 CaF2) on Page 5-6 and Page 24-25:

*‘PL difference observed in crystals grown on different substrates, as well as the PL inhomogeneity in one single crystal is attributed to the varying strain distribution. Electron backscatter diffraction (EBSD) is used to verify the strain by examining the relative orientation between perovskite and CaF2 substrate, as shown in Fig. 2c-e. The perovskite flowers show a single-crystalline character with <110> orientation close to z-axis and the <001> orientation close to the y-axis, according to the related pole figure for three different positions on the crystal (Fig. 2d & Supplementary Figure 10). CaF2 shows <111> orientation close to z-axis and <110> orientation close to y-axis. Combined with the lattice constant, the side view of the lattice-mismatched interface with well-defined relative orientation is depicted in Figure 2e. We reason that interfacial energy minimization is accompanied by the crystal deformation to reduce the interfacial mismatch, which could lead to strain inhomogeneity in the crystal. The complete pole figure and analysis can be found in Supplementary Figure 10 and Method.’*

10

**Figure 2. Hyperspectral and EBSD characterization for 35%Br crystals on CaF2 substrate. a** Wide-field optical images of 35%Br perovskite crystals fabricated on SrCaF2 single-crystal substrates *via* drop-casting method. The images were obtained under 50× object lens in reflection mode. **b** Wide-field hyperspectral images of perovskite crystals fabricated on SrCaF2 single-crystal substrates. 532 nm diode continuous-wave laser is used to excite the sample with approx. 100 mW cm^-2^ intensity. This image is constructed by superposing three narrow-band PL intensity mapping at 680 nm, 710 nm and 740 nm (5 nm bandwidth). The PL mapping at 680 nm, 710 nm and 740 nm are marked as blue, green and red color, respectively. The initial PL peak is located at around 700 nm and red-shift to 740nm after halide segregation. **c** Electron backscatter image (70° tilted view) for EBSD analysis on perovskite crystals of 35%Br fabricated on CaF2 substrate. Three different positions are characterized, marked by 1/2/3. **d** Pole figures of the substrate CaF2 and 35%Br perovskite derived from Kikuchi patterns of EBSD. Scale bar: 10 μm. **e** Side-view crystal structures of CaF2/35%Br perovskite oriented at <111>/<110> in z-axis and <110>/<001> in y-axis, respectively. Scale bar: 10 μm.

**a	CaF_2_**

**b**

z

**c**

**e**

y

y

x

**①**

**③**

**I Pb Ca F**

**②**

**(111)**

**(110)**

**d**

CaF_2_ <111>

MAPb(I0.65Br0.35)3 <110>

11

**Figure R3 a** SEM micrograph (70° tilted view) of free-standing single crystal 35%Br perovskite with indicated region for subsequent EBSD analysis. **b** Kikuchi pattern of 35%Br single crystal as derived . **c** Kikuchi pattern of on-CaF2 35%Br single crystal. **d** Pole figure of on-CaF235%Br crystal. Note in Figure d: from the Kikuchi pattern, it is almost impossible to distinguish between (110) and (002) lattice planes due to a high similarity between the patterns of the two planes, which is an intrinsic and recognized character for tetragonal perovskite phase. **e** Pole figure of the CaF2 substrate. **f** Top-view crystal structure of perovskite and the substrate CaF2. Top left cuboid represents the unit-cell orientation of tetragonal perovskite. Bottom left cubic represents the unit-cell orientation of cubic structure CaF2. The sample is 35%Br perovskite, and the CIF file of MAPbI3 is used here considering the same crystal structure.

4. (Fig. 3b,d) The symbols chosen do not allow a clear reading of the figure. It might help to use open symbols or semitransparent symbols.

**Response:**

We thank the reviewer for this warm-hearted suggestion and we made the revision in **Figure** 3.

**a	b**

Dark
Illumination
Dark recovery

**Intensity (a.u.)**

35%Br Film

Dark Illumination Dark recovery

**FWHM (Degree)**

**0.9**

**0.6**

**0.3**

**1.5**

**1.2**

**26	28	30	32	34**

**2θ (Degree)**

**c**

**Intensity (a.u.)**

**d**

65%Br Film

Dark Illumination Dark recovery

**FWHM (Degree)**

**0.9**

**0.6**

**1.5**

**1.2**

**0.3**

Dark Illumination Dark recovery

| **26 28 30 32 34**  **2θ (Degree)** |  |
| --- | --- |

5. It is not clear to this reviewer how the authors accommodate in their model apparently irreversible changes observed in PL under illumination (Fig. 4) at widely absent or reversible changes in XRD. A more explicit discussion would help.

**Response:**

We feel sorry for not clarifying that local PL characterization (**Fig**. 4) was implemented under **continuous laser illumination**, whereas the ‘Dark recovery’ in XRD (**Fig**. 3) denotes the sample

12

after **approx. 3 minutes stored under dark**. As discussed in **Response 2:** PL change is also reversible once the samples were stored under dark for a while. The reversibility of LHS has also been discussed by Richard H. Friend, Michael D. McGehee, Masaru Kuno *et al.* ^5,8,10.^ In our revised manuscript, we supplemented the measurement condition and the related discussion on the reversible change. On Page 6:

*‘All the peaks recovered to their original positions when the samples were stored in dark for approx. 2 minutes. The reversibility of XRD patterns is consistent with the reversible PL change* ***(Fig. S4****) 10,11.’*

*‘The XRD of dark recovery indicates the sample was stored under dark for around 3 minutes after the LED light was turned off.’*

6. (p.9 1st para) It should be pointed out that the presence of strain cannot be considered the only possible origin of peak broadening in XRD. The presence of structural defects and the size of uniformly scattering domains (effective crystal size according to Scherrer´s equation) can have similar consequences. Did the authors also apply this analysis to the data in Fig. 3a ?

**Response:**

As the reviewer mentioned, the broadening in XRD is related to many factors. Here in our manuscript, we assume that the broadening mainly contains three parts: instrumental effect, size effect, and strain effect. It’s true that the structural defects like Schottky defect should also be included; however, it turns out that they do not greatly affect the XRD broadening, because the derived strain value under our assumption is close to Williamson-Hall analysis that is based on the **proportionality** between *Bcos(θ)* of *sin(θ)*, where B and *θ* indicate the peak broadening and peak position.

We appreciate the reviewer’s careful reading of our manuscript for raising the last question. In **Figure** 5, we only showed the strain analysis for 65%/75%Br samples, not 35%Br sample, because the crystal structure of 35%Br perovskite is tetragonal leading to an intrinsic XRD splitting at plane (100) (110) (111), *etc.* ^12^ The splitting is discernable for MAPbI3 while they are overlapped for 35%Br perovskite. This overlap-induced broadening leads to invalid Williamson-Hall analysis. Here we show the Williamson-Hall plot of **35%Br poly-crystal film** below and present Williamson-Hall plot of **35%Br single crystal** in **Fig. S15**.

13

**a	b**

**Figure R4 a** Williamson-Hall plot of perovskite poly-crystal of 35%Br; **b** Williamson-Hall plot of single-crystal 35%Br.

**7.** (p9 2nd para) Raman data for MAPbI3 do not assist the discussion of the present changes. Data for Br0.35 would be helpful, but those for MAPbI3 can be omitted.

**Response:**

We thank the reviewer for raising this professional and insightful question. We agree with the reviewer that the micro-Raman data for 35%Br would be more convincing than that for MAPbI3 film.

However, the laser source of our confocal micro-Raman is 488 nm laser, which will induce an inhomogeneous LHS in 35%Br film during Raman measurement. This inhomogeneous LHS could induce an excess local strain at Br-rich/Br-poor interfaces^5^, which would lead to misinterpretation of strain inhomogeneity due to halide segregation for 35%Br sample.

In our humble opinion, the local strain in MAPbI3 polycrystalline film should be very similar to that in 35%Br film because of the same crystal structure and film morphology. Therefore, we present the Raman data for MAPbI3 to evidence the local strain inhomogeneity in perovskite film. In the revised manuscript, we added the figure of Raman variation for MAPbI3 polycrystalline film to **Figure** 5, on Page 11:

*‘we use confocal Raman to analyze the strain inhomogeneity in MAPbI3 film excited by a 488 nm laser, which gives an optical resolution of approximately 300 nm. Note that micro-Raman spectroscopy is sensitive to the local strain ^13,14.^ We select MAPbI3 film instead of 35%Br film because the 488 nm laser induces inhomogeneous LHS in 35%Br film. LHS itself has been reported to increase the strain at the phase-segregated regions^5^, giving rise to artifacts when analyzing the strain-inhomogeneity..’*

14

**Figure 5. Strain analysis in mixed-halide perovskites. a** Williamson-Hall plot of perovskite thin film of 65% and 75%Br fabricated through antisolvent method, in which _Bcorr._ represents the broadening corrected by instrumental broadening using X’Pert HighScore Plus software. **b** Relative strain of perovskite film to the single crystal of 65% and 75%Br using the formula (1). **c** Raman spectra of the decomposition process of MAPbI3 film during Raman measurement at 20°C. The integration time is 10 s for each test and the time interval in between is 0.1 s. Note that there is no Raman signal from the fresh perovskite before degradation. 488 nm CW laser is used here. **d** Raman spectra at different positions (1-5) in thin-film MAPbI3 after 20s laser illumination at 20°C. Note that we selected MAPbI3 instead of 35%Br to perform Raman test because 35%Br perovskites have inhomogeneous LHS during Raman measurement under 488 nm CW laser.

Very recently, Prof. David Ginger *et al.* proved the strain inhomogeneity in perovskite film using state-of-the-art EBSD^9^. Particularly, they evidenced that the strain is mainly located at the grain boundary in perovskite thin film, which coincidently provides strong evidence for our claim.

8. (p.11) While the passivation of LHS by KI or PEAI is impressive, it may have to be discussed in more detail. A grain size detected in SEM does not necessarily reflect the size of ordered domains effective in XRD. Less defects in the crystal lattice could also lead to more narrow XRD peaks. Strain-release, therefore, may of course play a role but at this point it cannot be considered proven.

**Response:**

We thank the referee’s concern about KI/PEAI’s effects on LHS.

15

Regarding the size effect, we admit that SEM images indeed do not reflect the size of the ordered grain, as demonstrated by David S. Ginger *et al.* using electron backscatter diffraction ^9.^ However, the size effect only contributes to approx. 0.02^o^ reduction even if we assume the size increases from 200 nm to 500 nm; this value is still lower than 0.07^o^ reduction due to KI additives.

For defects, although Samuel D. Stranks *et al.* reported the defect passivation’s effect of potassium on _(Cs,FA,MA)Pb(I0.85Br0.15)3,15_ we didn’t observe the same trend in the MAPbI*x*Br*3-x* system (**Fig. R6**).

In our opinion, the strain release might be the main reason for explaining the suppressed LHS. However, we **didn’t exclude the defect passivation’s effect on LHS** due to the fact that ion migration is always involved in LHS. On Page 11:

*‘The reduced broadening cannot be explained by size effect from the SEM images of perovskites with and without K! additive (Supplementary Figure 14). Even if we assume the grain size increases from 200 nm to 500 nm, it only contributes to approx. 0.02^o^ reduction, which is smaller than the total reduction of 0.07^o^. PL spectra do not strongly support the scenario of defects passivation of K! in our case of MAPb!xBr3-x system (Supplementary Figure 20). We, therefore, summarize that in addition to defects passivation26,46, strain release is also important to stabilize mixed-halide perovskites.’*

**35%Br with 20 mol.% KI**

**600	650	700	750	800**

**Intensity (a.u.)**

**35%Br control	b**

**a**

**3000**

**2000**

**1000**

**Intensity (a.u.)**

**600	650	700	750	800**

**3000**

**2000**

**1000**

**Wavelength (nm)	Wavelength (nm)**

**Figure R6 a.** PL spectra of the 35%Br perovskite film without KI (**a**) and with KI (**b**), under the same condition (laser intensity and detection distance). Different color in each figure indicates the initial PL spectra from varying position in one sample.

9. (p12 l.264) Since iodide and bromide do not form a separate phase, it seems more accurate to speak of a statistical distribution of the two within the perovskite crystals rather than speaking of an alloy.

**Response:**

We thank the referee’s suggestion and we revised ‘alloy’ to ‘mix’ through the whole manuscript.

16

1	Chen, Y. *et al.* Strain engineering and epitaxial stabilization of halide perovskites. *Nature* **577**, 209-215, doi:10.1038/s41586-019-1868-x (2020).

2	Fleck, N. A., Muller, G. M., Ashby, M. F. & Hutchinson, J. W. Strain Gradient Plasticity: Theory and Experiment. *Acta metall, mater.* **42**, 475-487 (1994).

3	Prieto;, J. E. & Markov, I. Step energies and equilibrium shape of strained monolayer islands. *Europhysics Letters* **108**, 46007 (2014).

4	Chen, Q. *et al.* Multiple-Stage Structure Transformation of Organic-Inorganic Hybrid Perovskite CH3NH3PbI3. *Physical Review X* **6**, 031042 doi:10.1103/PhysRevX.6.031042 (2016).

5	Hoke, E. T. *et al.* Reversible photo-induced trap formation in mixed-halide hybrid perovskites for photovoltaics. *Chem. Sci.* **6**, 613-617, doi:10.1039/c4sc03141e (2015).

6	Prabhu, Y. T., Rao, K. V., Kumar, V. S. S. & Kumari, B. S. X-Ray Analysis by Williamson-Hall and Size-Strain Plot Methods of ZnO Nanoparticles with Fuel Variation. *World Journal of Nano Science and Engineering* **04**, 21-28, doi:10.4236/wjnse.2014.41004 (2014).

7	Freund;, L. B. & Suresh, S. Thin Film Materials: Stress, Defect Formation and Surface Evolution. *Cambridge University Press* **1**, 65-95 (2003).

8	Slotcavage, D. J., Karunadasa, H. I. & McGehee, M. D. Light-Induced Phase Segregation in Halide-Perovskite Absorbers. *ACS Energy Letters* **1**, 1199-1205, doi:10.1021/acsenergylett.6b00495 (2016).

9	Sarthak Jariwala *et al.* Local Crystal Misorientation Influences Non-Radiative Recombination. *Joule* **3**, 3048-3060, doi[:](https://doi.org/10.1016/j.joule.2019.11.001)https://doi.org/10.1016/j.joule.2019.11.001 (2019).

10	Brennan, M. C., Draguta, S., Kamat, P. V. & Kuno, M. Light-Induced Anion Phase Segregation in Mixed Halide Perovskites. *ACS Energy Letters* **3**, 204-213, doi:10.1021/acsenergylett.7b01151 (2017).

11	Zhao, Y. *et al.* Quantification of light-enhanced ionic transport in lead iodide perovskite thin films and its solar cell applications. *Light: Science & Applications* **6**, e16243, doi:10.1038/lsa.2016.243 (2016).

12	Baikie, T. *et al.* Synthesis and crystal chemistry of the hybrid perovskite (CH3NH3)PbI3 for solid-state sensitised solar cell applications. *Journal of Materials Chemistry A* **1**, 5628, doi:10.1039/c3ta10518k (2013).

13	Neumann, C. *et al.* Raman spectroscopy as probe of nanometre-scale strain variations in graphene. *Nature communications* **6**, 8429, doi:10.1038/ncomms9429 (2015).

14	Veber, A., Cicconi, M. R., Reinfelder, H. & de Ligny, D. Combined Differential scanning calorimetry, Raman and Brillouin spectroscopies: A multiscale approach for materials investigation. *Anal Chim Acta* **998**, 37-44, doi:10.1016/j.aca.2017.09.045 (2018).

15	Abdi-Jalebi, M. *et al.* Maximizing and stabilizing luminescence from halide perovskites with potassium passivation. *Nature* **555**, 497-501, doi:10.1038/nature25989 (2018).

**Reviewer comments, third version:**

Reviewer #1 (Remarks to the Author):

This reviewer appreciates the additional efforts that the authors invested to restructure their manuscript and to explicitly include additional arguments in their discussion. Now, the paper is ready for publication, presenting all the valuable experimental findings in a way that the readership can fully utilize the outcome of the study. Good job !


Reviewer #2 (Remarks to the Author):

The authors have made substantial changes to the manuscript. The dataset is now more completed with more experimental work done, and the discussions is more in-depth. In spite of the efforts, I am afraid that in my opinion the manuscript it not up to the high standards of Nature Communications yet. The main deficiency is that the conclusions of this work were not properly drawn. The argument about strain-activated phase segregation was mostly based on a phenomenolgical study. The authors observed that the strain, either in the bulk or at the surfaces of the mixed-halide perovskite, was having an effect on the phase segregation phenomena. The mechanism, however, remained elusive. Moreover, the connection between optical illumination and the effects of strains was not discussed. Both of them were apparently playing roles in phase segregation. What was also missing in the study was the consistency or the conflict of this strain oriented mechanism to the ones reported previously. Such discussions would have been essential to advance the understandings of phase segregation in mixed-halide perovskites, rather than making another hypothesis of the underlying mechanism.

**Author rebuttal, third version:**

**Reviewer 2:**

The authors have made substantial changes to the manuscript. The dataset is now more completed with more experimental work done, and the discussions is more in-depth. In spite of the efforts, I am afraid that in my opinion the manuscript it not up to the high standards of Nature Communications yet. The main deficiency is that the conclusions of this work were not properly drawn. The argument about strain-activated phase segregation was mostly based on a phenomenolgical study. The authors observed that the strain, either in the bulk or at the surfaces of the mixed-halide perovskite, was having an effect on the phase segregation phenomena. The mechanism, however, remained elusive. Moreover, the connection between optical illumination and the effects of strains was not discussed. Both of them were apparently playing roles in phase segregation. What was also missing in the study was the consistency or the conflict of this strain oriented mechanism to the ones reported previously. Such discussions would have been essential to advance the understandings of phase segregation in mixed-halide perovskites, rather than making another hypothesis of the underlying mechanism.

**Response:**

We sincerely appreciate your time for giving these valuable comments to us in improving this work. To make sure we understand your concerns correctly, we summarized your questions into two aspects.

1. What is the mechanism of strain-activated halide segregation and the interplay between optical illumination and strain in halide segregation?

As is known, light-induced halide segregation (LHS) is a commonly recognized phenomenon in the community of mixed-halide perovskites. The microscopic mechanisms of LHS itself have already been rationalized by bandgap reduction of iodide-rich phases and polaronic effect under light illumination^1,2^. However, most studies were based on polycrystalline films, rendering them to conclude that LHS is exclusively an intrinsic property in mixed-halide perovskites.

Our study corrects the above conclusion by identifying strain-activated LHS. The most important perovskites with bandgap of approx. 1.7 eV are within the regime of strain-activated LHS. That is, without strain or lattice deformation in perovskite, the driving force of bandgap reduction and polaronic effect is not enough to induce the halide segregation under optical excitation. From the free energy point of view

Without lattice deformation:

$$\Delta F=F_{seg.}-F_{mix}$$

$$=\left( U_{seg.}-U_{mix} \right)-k_{B}TN_{0}\left[ x_{\mathrm{Br}}lnx_{\mathrm{Br}}+\left( 1-x_{\mathrm{Br}} \right)ln \left( 1-x_{\mathrm{Br}} \right) \right]+N{\Delta E}_{g}+N {\Delta E}_{p}+(E_{seg.}^{s1.}-E_{mix}^{s1.})$$

$$=\Delta U-T\Delta S+N{\Delta E}_{g}+N {\Delta E}_{p}+(E_{seg.}^{s1.}-0)$$

With lattice deformation:

$$\Delta F=F_{seg.}-F_{mix}$$

$$=\left( U_{seg.}-U_{mix} \right)-k_{B}TN_{0}\left[ x_{\mathrm{Br}}lnx_{\mathrm{Br}}+\left( 1-x_{\mathrm{Br}} \right)ln \left( 1-x_{\mathrm{Br}} \right) \right]+N{\Delta E}_{g}+N {\Delta E}_{p}+(E_{seg.}^{s2.}-E_{mix}^{s2.})$$

$$=\Delta U-T\Delta S+N{\Delta E}_{g}+N {\Delta E}_{p}+\Delta E^{s}$$

From experimental results of the bandgap against *x*_Br_:

$$\Delta E_{g}=(0.9x_{\mathrm{Br}}-0.25x_{\mathrm{Br}}^{2})\cdot N$$

Where U, S, T is formation energy of perovskite, entropy, temperature respectively; $N$ is carrier density, ${\Delta E}_{g}$ is the energy reduction by the funneling of photo-carriers to I-rich clusters; ${\Delta E}_{p}$is the reduction of polaronic energy due to halide segregation, $\Delta E^{s}$ is the gain of strain energy around I-rich/Br-rich interfaces due to halide segregation. For$\Delta F$ < 0, the halide segregation state is more favored than the mixed state.

As a function of Br concentration (*x*_Br_), the sum of the first two terms is positive, while the third and the fourth term is negative for *x*_Br_ > 20% *^1,2^*. The last term is positive considering the lattice mismatch between I-rich and Br-rich perovskite. Without lattice deformation (*e.g.* free-standing single crystals), the strain energy is close to 0 at the mixed state; however, it becomes non-trivial for the strained perovskites at the mixed state.

The halide segregation will occur when $\boldsymbol{\Delta F<0}$**.** Only the 3^rd^ and the 4^th^ term are the driving force of halide segregation because the photo-carriers can funnel into the iodide-rich clusters to minimize the total free energy. We can derive that the gain of strain energy caused by halide segregation is lower for the strained perovskite ($\Delta E^{s2}=E_{seg.}^{s2.}-E_{mix}^{s2.}$), compared with the unstrained perovskite ($E_{seg.}^{s1.}$). Thus, a lower driving force is needed for the strained perovskites to induce halide segregation.

We believe that a related theoretical simulation on the synergetic effect of strain and light will be more insightful, but it involves excited-state theory and complex lattice deformation. This requires a huge effort and deserves another independent study. To properly conclude, we supplement a deeper discussion in the revised manuscript (Page 11 & Supplementary Note 3)

*‘’In terms of thermodynamic phase stability under dark, Br can be stably mixed with iodide in the whole composition space of MAPb(I_1-x_Br_x_)_3_. Under optical illumination, the formation of iodide-rich phases is observed, which is rationalized by bandgap reduction and polaronic effect^1,2^. Although the light-induced halide segregation is commonly recognized, it is still regarded as an intrinsic property in mixed-halide perovskites ^3-11^. Our results elaborate on the incomplete understanding of LHS by unveiling the effects of strain on LHS. From the free energy point of view, it demands a larger driving force for the unstrained perovskite to induce halide segregation (****Supplementary Note 3****). Thus, two distinct regimes should be recognized: strain-activated LHS regime and intrinsic-LHS regime. We conclude that, without the synergetic effect of strain, the driving force of bandgap reduction and polaron is not enough to induce halide segregation within the strain-activated LHS regime.’’*

**Supplementary Note 3**

***‘’****To discuss the mechanism of strian-activated LHS, we use a simplified model to analyze the thermodynamics of mixed-halide perovskite with and without lattice deformation, or internal strain, as follows:*

*Without lattice deformation:*

$$\Delta F=F_{seg.}-F_{mix}$$

$$=\left( U_{seg.}-U_{mix} \right)-k_{B}TN_{0}\left[ x_{Br}lnx_{Br}+\left( 1-x_{Br} \right)ln \left( 1-x_{Br} \right) \right]+N{\Delta E}_{g}+N {\Delta E}_{p}+(E_{seg.}^{s1.}-E_{mix}^{s1.})$$

$$=\Delta U-T\Delta S+N{\Delta E}_{g}+N {\Delta E}_{p}+(E_{seg.}^{s1.}-0)$$

*With lattice deformation:*

$$\Delta F=F_{seg.}-F_{mix}$$

$$=\left( U_{seg.}-U_{mix} \right)-k_{B}TN_{0}\left[ x_{Br}lnx_{Br}+\left( 1-x_{Br} \right)ln \left( 1-x_{Br} \right) \right]+N{\Delta E}_{g}+N {\Delta E}_{p}+(E_{seg.}^{s2.}-E_{mix}^{s2.})$$

$$=\Delta U-T\Delta S+N{\Delta E}_{g}+N {\Delta E}_{p}+\Delta E^{s2}$$

*From experimental results:*

$$\Delta E_{g}=(0.9x_{Br}-0.25x_{Br}^{2})\cdot N$$

*Where U, S, T is formation energy of perovskite, entropy, temperature respectively;* $N$*_0_ is lattice cell number;* $N$ *is carrier density,* ${\Delta E}_{g}$ *is the energy reduction by the funneling of photo-carriers to I-rich clusters;* ${\Delta E}_{p}$*is the reduction of polaronic energy due to halide segregation,* $\Delta E^{s}$ *is the gain of strain energy around I-rich/Br-rich interfaces due to halide segregation.*

*As a function of Br concentration (x_Br_), the sum of the first two terms is positive, while the third and the fourth term is negative for x_Br_ > 20% ^1,2^. The last term is positive considering the lattice mismatch between I-rich and Br-rich perovskite. Without lattice deformation (e.g. free-standing single crystals), the strain energy is close to 0 at the mixed state; however, it becomes non-trivial for the strained perovskites at the mixed state.*

*The halide segregation will occur if* $\boldsymbol{\Delta F<0}$***.*** *Only the 3^rd^ and the 4^th^ term are the driving force of halide segregation because the photo-carriers can funnel into the iodide-rich clusters to minimize the total free energy. We can derive that the gain of strain energy caused by halide segregation is lower for the strained perovskite (*$\Delta E^{s2}=E_{seg.}^{s2.}-E_{mix}^{s2.}$*), compared with the unstrained perovskite (*$E_{seg.}^{s1.}$*). Thus, it is equivalent to a lower driving force for the strained perovskites to induce halide segregation****.’’***

2. What is the consistency or the conflict of this strain oriented mechanism to the ones reported previously?

Previous studies utilized static pressure *via* diamond-anvil cell to study the pressure’s effects on LHS for polycrystalline films or powders ^12,13^. In the liquid cell, an isotropic lattice compression is induced in perovskites by applying external pressure to the liquid inside. Interestingly, they observed that LHS is suppressed under high pressure of approx. 1GPa, which is attributed to the suppressed ion migration in the strongly compressed lattice. We think our results are not contradictory, but complementary with their conclusion.

First, our experiments are performed with uniaxial pressure, leading to compression in one axis while expansion in the orthogonal axis considering a positive Poisson's ratio. Second, our single crystals do not show LHS while the polycrystalline samples in previous studies normally suffer LHS with the same composition (40%Br). Thus, we can conclude that the LHS in previous experiments is only kinetically suppressed by inhibiting the ion migration (the more compact of lattice, the less migration of halide), which overwhelms the tendency of LHS. Based on previous reports^12,13^, we might be able to further conclude that tensile strain, not the compressive strain is the key to activate LHS in the single crystals or the polycrystalline films. Thanks to the reviewer’s insightful suggestion, we supplement the relevant discussion in the revised manuscript with an upgraded schematic in Fig. 1 (Page 3)

*‘’Previous studies of pressure’s effect on LHS are based on diamond-anvil cells to induce isotropic lattice compression in perovskite powders^12,13^. The LHS is found to be kinetically suppressed due to inhibited ion migration (the more compact of lattice, the less migration of halide). In contrast, here we applied uniaxial pressure to the single crystal, resulting in compression and expansion of the lattice in two orthogonal axes considering a positive Poisson's ratio. Therefore, it is derived that the tensile strain or complex lattice deformation, not the compressive strain may be the key to activate LHS in the single crystals.’’*

We hope you find that this manuscript now presents more thoroughly the connection between optical illumination and strain, as well as the correlation with previous studies. Again, thank you so much for your expertise and valuable time in reviewing this work.

References:

1	Draguta, S. *et al.* Rationalizing the light-induced phase separation of mixed halide organic-inorganic perovskites. *Nature communications* **8**, 200, doi:10.1038/s41467-017-00284-2 (2017).

2	Bischak, C. G. *et al.* Origin of Reversible Photoinduced Phase Separation in Hybrid Perovskites. *Nano letters* **17**, 1028-1033, doi:10.1021/acs.nanolett.6b04453 (2017).

3	Tang, X. *et al.* Local Observation of Phase Segregation in Mixed-Halide Perovskite. *Nano letters* **18**, 2172-2178, doi:10.1021/acs.nanolett.8b00505 (2018).

4	Mao, W. *et al.* Visualizing Phase Segregation in Mixed-Halide Perovskite Single Crystals. *Angewandte Chemie* **58**, 2893-2898, doi:10.1002/anie.201810193 (2019).

5	Chen, W., Mao, W., Bach, U., Jia, B. & Wen, X. Tracking Dynamic Phase Segregation in Mixed‐Halide Perovskite Single Crystals under Two‐Photon Scanning Laser Illumination. *Small Methods*, 1900273, doi:10.1002/smtd.201900273 (2019).

6	Gualdron-Reyes, A. F. *et al.* Controlling the Phase Segregation in Mixed Halide Perovskites through Nanocrystal Size. *ACS Energy Lett* **4**, 54-62, doi:10.1021/acsenergylett.8b02207 (2019).

7	Wang, X. *et al.* Suppressed phase separation of mixed-halide perovskites confined in endotaxial matrices. *Nature communications* **10**, 695, doi:10.1038/s41467-019-08610-6 (2019).

8	Belisle, R. A. *et al.* Impact of Surfaces on Photoinduced Halide Segregation in Mixed-Halide Perovskites. *ACS Energy Letters* **3**, 2694-2700, doi:10.1021/acsenergylett.8b01562 (2018).

9	Levchuk, I. *et al.* Brightly Luminescent and Color-Tunable Formamidinium Lead Halide Perovskite FAPbX3 (X = Cl, Br, I) Colloidal Nanocrystals. *Nano letters* **17**, 2765-2770, doi:10.1021/acs.nanolett.6b04781 (2017).

10	Xiao, Z. *et al.* Mixed-Halide Perovskites with Stabilized Bandgaps. *Nano letters* **17**, 6863-6869, doi:10.1021/acs.nanolett.7b03179 (2017).

11	Hoke, E. T. *et al.* Reversible photo-induced trap formation in mixed-halide hybrid perovskites for photovoltaics. *Chem. Sci.* **6**, 613-617, doi:10.1039/c4sc03141e (2015).

12	Slotcavage, D. J., Karunadasa, H. I. & McGehee, M. D. Light-Induced Phase Segregation in Halide-Perovskite Absorbers. *ACS Energy Letters* **1**, 1199-1205, doi:10.1021/acsenergylett.6b00495 (2016).

13	Jaffe, A. *et al.* High-Pressure Single-Crystal Structures of 3D Lead-Halide Hybrid Perovskites and Pressure Effects on their Electronic and Optical Properties. *ACS Cent Sci* **2**, 201-209, doi:10.1021/acscentsci.6b00055 (2016).
